# Understanding the challenges to COVID-19 vaccines and treatment options, herd immunity and probability of reinfection

**DOI:** 10.1016/j.jtumed.2022.11.007

**Published:** 2022-12-17

**Authors:** Mohammad A.I. Al-Hatamleh, Mai A. Abusalah, Ma'mon M. Hatmal, Walhan Alshaer, Suhana Ahmad, Manali H. Mohd-Zahid, Engku Nur Syafirah E.A. Rahman, Chan Y. Yean, Iskandar Z. Alias, Vuk Uskoković, Rohimah Mohamud

**Affiliations:** a Department of Immunology, School of Medical Sciences, Universiti Sains Malaysia, Kubang Kerian, Malaysia; b Department of Medical Laboratory Sciences, Faculty of Allied Medical Sciences, Zarqa University, Zarqa, Jordan; c Department of Medical Laboratory Sciences, Faculty of Applied Medical Sciences, The Hashemite University, Zarqa, Jordan; d Cell Therapy Center (CTC), The University of Jordan, Amman, Jordan; e Department of Chemical Pathology, School of Medical Sciences, Universiti Sains Malaysia, Kubang Kerian, Malaysia; f Department of Microbiology and Parasitology, School of Medical Sciences, Universiti Sains Malaysia, Kubang Kerian, Malaysia; g TardigradeNano LLC, Irvine, CA, USA

**Keywords:** سارس, كوفيد-19, فيروس كورونا, عودة العدوى, علاج البلازما, مناعة القطيع, 2019-nCoV, Convalescent plasma therapy, Coronavirus, Herd immunity, Reinfection, SARS-CoV-2

## Abstract

Unlike pandemics in the past, the outbreak of coronavirus disease 2019 (COVID-19), which rapidly spread worldwide, was met with a different approach to control and measures implemented across affected countries. The lack of understanding of the fundamental nature of the outbreak continues to make COVID-19 challenging to manage for both healthcare practitioners and the scientific community. Challenges to vaccine development and evaluation, current therapeutic options, convalescent plasma therapy, herd immunity, and the emergence of reinfection and new variants remain the major obstacles to combating COVID-19. This review discusses these challenges in the management of COVID-19 at length and highlights the mechanisms needed to provide better understanding of this pandemic.

## الملخص

على عكس الأوبئة في الماضي، شهد تفشي مرض كوفيد-19 الذي ينتقل بسرعة في جميع أنحاء العالم نهجا مختلفا تماما للضوابط والتدابير التي تم تنفيذها في جميع البلدان المتضررة. لا يزال عدم فهم الطبيعة الأساسية لتفشي المرض يجعل من كوفيد-19 تحديا لكل من ممارسي الرعاية الصحية والمجتمع العلمي. لا تزال التحديات التي تواجه تطوير اللقاح وتقييمه، والخيارات العلاجية الحصرية، وعلاج البلازما في فترة النقاهة، ومناعة القطيع، فضلا عن ظهور العدوى مرة أخرى والمتغيرات الجديدة، هي العقبات الرئيسية أمام مكافحة كوفيد-19. تناقش هذه المراجعة هذه التحديات في إدارة كوفيد-19 بشكل مفصل وتسلط الضوء على الآليات اللازمة لمساعدتنا على فهم هذا الوباء بشكل أفضل.

## Introduction

**Figure 1:**
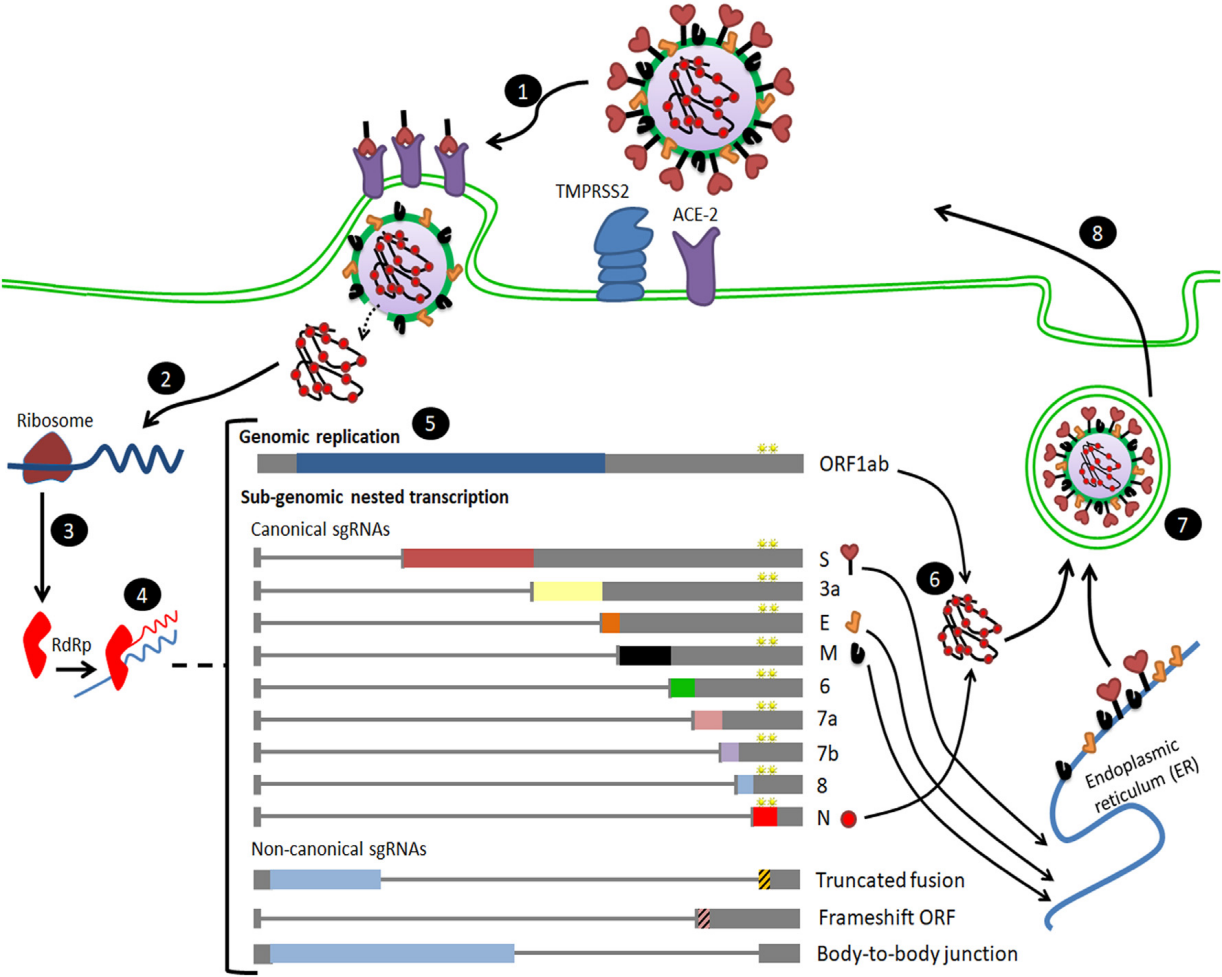
Structure, cell entry and replication mechanisms of SARS-CoV-2. The SARS-CoV-2 spike (S) protein binds angiotensin-converting enzyme 2 (ACE2). The transmembrane protease serine 2 (TMPRSS2) protease enzyme cleaves the S protein, thus allowing viral entry into the host cell cytosol after fusion of the viral and cellular membranes (1). SARS-CoV-2 genomic RNA is translated into non-structural proteins (NSPs) from two open reading frames (ORF1a and ORF1b) (2). Some of the NSPs have RNA-dependent RNA polymerase (RdRp) activity (nsp12) (3). Negative-sense RNA intermediates are generated to serve as the templates for the synthesis of positive-sense genomic RNA (gRNA) (4) and sub-genomic RNAs (sgRNAs) (5). The gRNA is packaged by the structural proteins to assemble progeny virions. Shorter sgRNAs encode conserved structural proteins (S, E, M and N) and several accessory proteins. SARS-CoV-2 is known to have at least six accessory proteins (3a, 6, 7a, 7b, 8 and 10), and non-canonical sgRNAs are also shown in the subfigure (6). The AAGAA-type modification clusters in gRNA and sgRNAs are shown with yellow star annotations. Thereafter, budding and exocytosis of the virus occurs (7 and 8) (Adapted from Hatmal et al., 2020^3^).

Since it was first reported in Wuhan, China, in December 2019, communicable coronavirus disease 2019 (COVID-19) caused by severe acute respiratory syndrome coronavirus 2 (SARS-CoV-2) continues to impose health and socioeconomic threats globally. SARS-CoV-2 belongs to the coronavirus family but is different from previous human coronaviruses (e.g., SARS-CoV and Middle East respiratory syndrome CoV [MERS-CoV]) in that it results in disease progression that is difficult to predict and is varied.^1^ SARS-CoV-2 has a receptor domain with high affinity toward angiotensin-converting enzyme 2 (ACE-2), which is abundant in many organs (Figure 1), thus explaining the varying symptoms and severity of COVID-19.^2^^,^^3^

The medical community initially expected that reaching the vaccination stage would not be feasible for a very long time. Consequently, research studies and clinical trials were initiated to assess the potential effects of several existing drugs against COVID-19.^4^ Furthermore, owing to its extensive history of efficacy against human respiratory viral infections, convalescent plasma (CP) therapy was used to treat COVID-19.^5^ This treatment option was used primarily for severely ill patients hospitalized with COVID-19, and has been shown to be effective for reducing the mortality risk of COVID-19. However, given the unprecedented speed of community transmission, the CP therapy did not assisted in achieving herd immunity to COVID-19, which requires a sufficient number of people in a population to have developed protective antibodies against SARS-CoV-2 through either vaccination or natural infection. Hence, the world awaited COVID-19 vaccines that offered hope to defeat the COVID-19 pandemic.

Currently, six vaccines (Pfizer-BioNTech, Moderna, AstraZeneca-Oxford, Johnson & Johnson, Sputnik V, Sinopharm and Sinovac) are authorized and available for immunization against COVID-19. However, no evidence indicates that recovered people with existing SARS-CoV-2 antibodies are protected against a second infection of COVID-19,^6^ and a growing number of studies report COVID-19 reinfections.^7^, ^8^, ^9^ This comprehensive review provides a broad overview of the challenges associated with COVID-19 vaccines and treatment options, including the potential of CP therapy, herd immunity, and the probability of reinfection.

## SARS-CoV-2 variants

Since the early stages of the COVID-19 pandemic, multiple strains of SARS-CoV-2 have emerged and spread worldwide. In open-source platforms such as Pango,^10^ Nexstrain (3103 genome sequences),^11^ GISAID (10,298,748 genome sequences)^12^ and NCBI Virus (4,616,599 nucleotide sequences),^13^ more than 5 million genome sequences have been provided. According to their phylogenetic analyses, clades have been determined according to a collection of common mutations^14^ and the ancestral clade known as clade O.^15^ Other clades such as L, S and A2a, as well as clade G, have become more prevalent after 2020.^16^, ^17^, ^18^

According to the WHO,^19^ the European Centre for Disease Prevention and Control (ECDC)^20^ and Centers for Disease Control and Prevention (CDC),^21^ a classification system has been developed to differentiate variants of concern (VOCs), variants of interest (VOIs), variants under monitoring (VUM), variants being monitored (VBM), formerly monitored variants (FMV) and variants of high consequence (VOHC).^22^ After the emergence of variants that posed a greater risk to global public health in late 2020, characterization of specific VOCs, VUMs, VOIs and VOHCs became necessary to prioritize research and global monitoring, and to influence the continuing response to the COVID-19 pandemic.^19^

COVID-19 variants that meet specific criteria, such as high transmission or adverse alterations in COVID-19 distribution; changes in clinical illness presentation or elevated clinical virulence; diminished effectiveness of social interventions and public health measures; or diminished effectiveness of diagnostics, treatments and vaccinations are defined as VOCs.^19^^,^^23^ VOCs are mutant strains that are relatively more transmissible, elude antibody neutralization, induce more severe disease progression, confound identification and increase the incidence of mortality.^22^ Particular VOCs may have selective benefits, on the basis of the existence of the same mutations repeatedly emerging in many virus strains in different geographic locations (i.e., convergent evolution).^11^^,^^20^^,^^24^^,^^25^ Most VOCs studied since the SARS-CoV-2 pandemic have had relatively higher transmissibility and virulence; these were the Alpha, Delta, Gamma and Beta variants. Although Gamma, Alpha, and Beta are no longer designated as VOCs, studying the causes that led to their emergence in populations is crucial.^22^ According to the SARS-CoV-2 Interagency Group of the United States government, the Omicron variant is considered a VOC.^21^ VOCs include Delta (1.617.2) and Omicron (B.1.1.529, BA.1, BA.2, BA.3, BA.4, BA.5, XE [a recombinant of Omicron BA.1 and BA.2] and descendent lineages).^19^^,^^21^

In contrast, VOIs are characterized as genetically mutated variants with predicted or known effects on viral characteristics, such as disease severity, transmissibility, immunological breakout, and therapeutic or diagnostic escape.^23^ In addition, these variants are characterized by the presence of specific genetic markers that may be associated with high transmissibility or virulence, the ability to escape detection, a decrease in neutralizing antibodies (NAbs) acquired via natural infection or vaccination, or a decrease in the effectiveness of vaccination or therapeutics.^23^ Furthermore, these variants have been identified as causing substantial and unique COVID-19 outbreak clusters or community transmission in various countries, with corresponding increases in the prevalence rates or other clear epidemiological effects, thus indicating a rising risk to global public health.^19^ VOIs include Omicron (BA.2.75) and Omicron (BA.2 + L452X (x)).^20^

VUMs are viral variants that bear genetic modifications suspected to affect the characteristics of the virus and that show indications of future risk to human health.^19^ Moreover, these variants are associated with more severe disease or greater transmission, but are either no longer detectable or are circulating at extremely low levels. However, the evidence of epidemiological or phenotypic influence is now uncertain, thus necessitating increased monitoring and reassessment in the absence of additional evidence. A VOI or VOC may be relegated to this category if its national and regional percentages have decreased significantly and consistently over time, or if other evidence suggests that a variant does not constitute a major risk to public health.^21^ This category includes former VOIs/VOCs that will continue to be tracked for an extended length of time and will retain the WHO label assigned to them until further notice. Some evidence suggests that they may have characteristics identical to those of VOCs; however, the evidence is either insufficient or has not yet been evaluated by the ECDC. According to the ECDC, VUMs include Omicron (BA.3).^20^ The WHO has established a new category in its variant tracking system, designated “Omicron subvariants under monitoring,” to indicate to public health authorities worldwide which VOC lineages may warrant prioritization and monitoring, in light of the broad transmission of the Omicron VOC worldwide and the expected increase in its viral diversity. Omicron subvariants under monitoring include BA.4 and BA.5, as well as lineages that share the same cluster of mutations in the spike, namely BA.2.12.1, BA.2.9., BA.2.11, BA.2.13 and BA.2.75, and the following variations seen outside the spike: BA.4: ORF7b:L11F, N:P151S; BA.5: M:D3N; ORF1a:del141/143 and ORF6:D61L.^19^

Since September 2021, the ECDC has categorized some variants as de-escalated variants,^20^ although these variants had been classified as VUMs by the CDC and as VOIs and/or VOCs by the WHO.^19^^,^^21^ These variants meet at least one of the following criteria: (1) the variant has spread for a long period of time without causing any overall epidemiological effects; (2) the variant is no longer circulating; and (3) scientific research has demonstrated that the variant does not contain any concerning features. Recently, on the basis of ECDC advice, the de-escalated variants have come to include Alpha (B.1.1.7), Beta (B.1.351), Gamma (P.1), Epsilon (B.1.427 and B.1.429), Zeta (P.2), Eta (B.1.525), Theta (P.3), Iota (B.1.526), Kappa (B.1.617.1), B.1.640, Lambda (C.37) and Mu (B.1.621).^20^

In this section, we discuss the main COVID-19 variants along with their severity, transmissibility and immunity characteristics (Table 1). Although some SARS-CoV-2 variants (e.g., B.1.1.7, P.1 and 501Y. V2) have been identified to be more transmissible variants and are already spreading rapidly worldwide,^26^, ^27^, ^28^ Delta and Omicron currently have the highest transmissibility and virulence.^22^ Although Delta has dispersed worldwide, Omicron is growing at a faster rate than Delta, and it bears an increased risk of transmission, particularly among contacts outside the household.^29^^,^^30^ Consequently, although early indications have suggested that individuals may be less likely to require hospitalization, a much greater number of people are anticipated to become infected. Regular contact tracing analyses have revealed that transmission rates among people infected with BA.2 are expected to be higher (13.4%) than those among household contacts of other people infected with Omicron (10.3%).^30^ The higher transmissibility of these variants has been associated with the mutations that they carry, including mutations that alter their S proteins (particularly the receptor-binding domain), the main target of COVID-19 vaccines and therapeutics.^3^ These mutations may decrease the binding affinity of anti-SARS-CoV-2 antibodies.^31^ Therefore, the growing number of more transmissible SARS-CoV-2 variants may cause more reinfections. In addition, existing vaccines may require future updates to improve their efficacy, particularly if vaccine-resistant SARS-CoV-2 variants emerge.

**Table 1:** Main COVID-19 variants and their severity, transmissibility and immunity characteristics.

WHO label	Lineage (Pango)	GISAID clade	Other names	Most common countries (earliest date)	Status from September 2021	Effect on severity	Effect on transmissibility	Effect on immunity	References
Delta	B.1.617.2 (Indian variant)	G/478K.V1	VUI-21APR-02	USA 21.0%, India 18.0%, UK 13.0%, Turkey 11.0%, Germany 5.0%	WHO: previously circulating VOC CDC: VBM ECDC: de- escalated	Increased	Increased	Increased	32, 33, 34, 35, 36
Alpha	B.1.1.7 (British variant)	GRY	VOC 202012/01, 20I/501Y.V1	UK 24.0%, USA 20.0%, Germany 9.0%, Sweden 6.0%, Denmark 6.0%	WHO: previously circulating VOC CDC: VBM ECDC: de-escalated	Increased	Identical to previous variants in that it was unlikely to have an effect	Increased	37, 38, 39, 40
Beta	B.1.351 (South African variant)	GH/501Y.V2	20H/501Y.V2	South Africa 19.0%, Philippines 9.0%, USA 9.0%, Sweden 8.0%, Germany 7.0%	WHO: previously circulating VOC CDC: VBM ECDC: de-escalated	Increased	Increased	Increased	^41^,^40^, ^42^, ^43^, ^44^, ^45^
Gamma	P.1 (Brazilian variant)	GR/501Y.V3	20J/501Y.V3	Brazil 56.0%, USA 29.0%, Chile 3.0%, Argentina 2.0%, Spain 1.0%	WHO: previously circulating VOC CDC: VBM ECDC: de-escalated	Increased	Increased	Increased	^40^,^46^, ^47^, ^48^
Epsilon	B.1.427 and B.1.429 (Californian variant)	GH/452R.V1	CAL.20C/L452R	B.1.427: USA 98.0%, Mexico 1.0%, Aruba 0.0%, Argentina 0.0%, Canada 0.0% B.1.429: USA 97.0%, Canada 2.0%, Mexico 1.0%, South Korea 0.0%, Chile 0.0%	WHO: previously circulating VOI CDC: VBM ECDC: de-escalated	Unclear	Increased	No evidence	^20^,^49^, ^50^, ^51^
Eta	B.1.525	G/484K.V3	–	Canada 19.0%, USA 15.0%, Germany 9.0%, France 8.0%, Denmark 7.0%	WHO: previously circulating VOI CDC: VBM ECDC: de-escalated	No evidence	Increased	No evidence	^20^,^52^, ^53^, ^54^
Theta	P.3	GR/1092K.V1	–	Philippines 83.0%, USA 3.0%, Germany 2.0%, Malaysia 2.0%, UK 2.0%	WHO: previously circulating VOI CDC: N/A ECDC: de-escalated	Increased	Increased	No evidence	^38^,^53^
Kappa	B.1.617.1	G/452R.V3	–	India 72.0%, UK 6.0%, Canada 6.0%, USA 5.0%, Ireland 3.0%	WHO: previously circulating VOI CDC: VBM ECDC: de-escalated	Increased	Increased	No evidence	55, 56, 57, 58
Zeta	P.2	GR/484K.V2	VUI-202101/01, B.1.1.28.2	Brazil 57.0%, USA 24.0%, Canada 4.0%, Argentina 2.0%, Paraguay 2.0%	WHO: previously circulating VOI CDC: VBM ECDC: de-escalated	No evidence	Increased	No evidence	^20^,^53^,^59^
Lambda	C.37	GR/452Q.V1	–	Peru 44.0%, Chile 19.0%, Argentina 13.0%, USA 12.0%, Ecuador 3.0%	WHO: previously circulating VOI CDC: N/A ECDC: de-escalated	No evidence	Increased	No evidence	60, 61, 62
Mu	B.1.621	GH	–	USA 40.0%, Colombia 27.0%, Chile 8.0%, Spain 4.0%, Panama 4.0%	WHO: previously circulating VOI CDC: VBM ECDC: de-escalated	Increased	Increased	No evidence	^38^,^53^,^63^
Iota	B.1.526	GH/253G.V1	–	USA 97.0%, Ecuador 1.0%, Canada 1.0%, Puerto Rico 1.0%, Spain 0.0%	WHO: previously circulating VOI CDC: VBM ECDC: de-escalated	No evidence	Increased	No evidence	^20^,^53^,^64^
N/A	XD (France variant)	–	–	France 68.0%, Denmark 28.0%, Netherlands 4.0%	WHO: FMV CDC: N/A ECDC: de-escalated	No evidence	No evidence	No evidence	^20^,^65^
N/A	XE (British variant)	–	–	UK 99.0%, USA 1.0%, Ireland 0.0%, Denmark 0.0%	WHO: VOC CDC: N/A ECDC: N/A	No evidence	No evidence	No evidence	^20^^,^^66^
N/A	XF (British variant)	–	–	UK 100.0%	WHO: N/A CDC: N/A ECDC: de-escalated	No evidence	No evidence	No evidence	^20^,^67^
Omicron	BA.1 (B.1.1.529)	GR/484A	–	USA 38.0%, UK 29.0%, Canada 5.0%, Germany 4.0%, Norway 3.0%	WHO: VOC CDC: VOC ECDC: VOC	Increased	Increased	Decreased	68, 69, 70, 71, 72, 73, 74, 75
Omicron	BA.2 (B.1.1.529)	GR/484A	–	UK 67.0%, Germany 8.0%, USA 4.0%, Denmark 3.0%, France 3.0%	WHO: VOC CDC: VOC ECDC: VOC	Increased	Increased	Decreased	^69^,^76^, ^77^, ^78^, ^79^, ^80^
Omicron	BA.3(B.1.1.529)	GR/484A	–	UK 35.0%, Poland 24.0%, Germany 17.0%, South Africa 5.0%, USA 4.0%	WHO: VOC CDC: VOC ECDC: VUM	No evidence	No evidence	No evidence	^20^,^81^
Omicron	BA.4 (B.1.1.529)	GR/484A	–	UK 28.0%, USA 27.0%, South Africa 8.0%, Israel 5.0%, Denmark 5.0%	WHO: VOC CDC: VOC ECDC: VOC	No evidence	Increased	No evidence	^20^^,^^82^
Omicron	BA.5 (B.1.1.529)	GR/484A	–	USA 24.0%, UK 19.0%, Denmark 9.0%, France 5.0%, South Africa 5.0%	WHO: VOC CDC: VOC ECDC: VOC	No evidence	Increased	No evidence	^20^,^83^
Omicron	BA.2 + L452X	GR/484A	–	UK 32.0%, Germany 13.0%, Denmark 13.0%, USA 11.0%, France 5.0%	WHO: VOC CDC: VOC ECDC: VOI	No evidence	Increased	No evidence	^76^,^84^
Omicron	BA.2.75	GR/484A	–	India 76.0%, UK 8.0%, USA 4.0%, New Zealand 3.0%, Canada 2.0%	WHO: VOC CDC: VOC ECDC: VOI	No evidence	No evidence	No evidence	^84^,^85^

VOC, variants of concern; VOI, variants of interest; VUM, variants under monitoring; VBM, variant being monitored; FMV, formerly monitored variants; N/A, not applicable.

## Vaccine development against COVID-19

Vaccines are artificial or natural biological preparations used to stimulate the immune response for antibody production against a particular disease. A vaccine typically consists of a pathogenic agent (either the microorganism itself, or its specific antigen epitope, surface protein, toxin or a synthetic substitute) treated to act as an antigen without causing disease in a host.^86^ A vaccine works by “educating” the immune system to recognize and fight against microbial pathogens, i.e., either viruses or bacteria. Thus, a particular molecule (e.g., antigen epitope, surface protein or toxin) from the pathogen must be presented to the host to induce an immune response.^87^ The production of antibodies from the given antigen provides immunity, which helps the immune system become stronger when it encounters a real infection.

**Figure 2:**
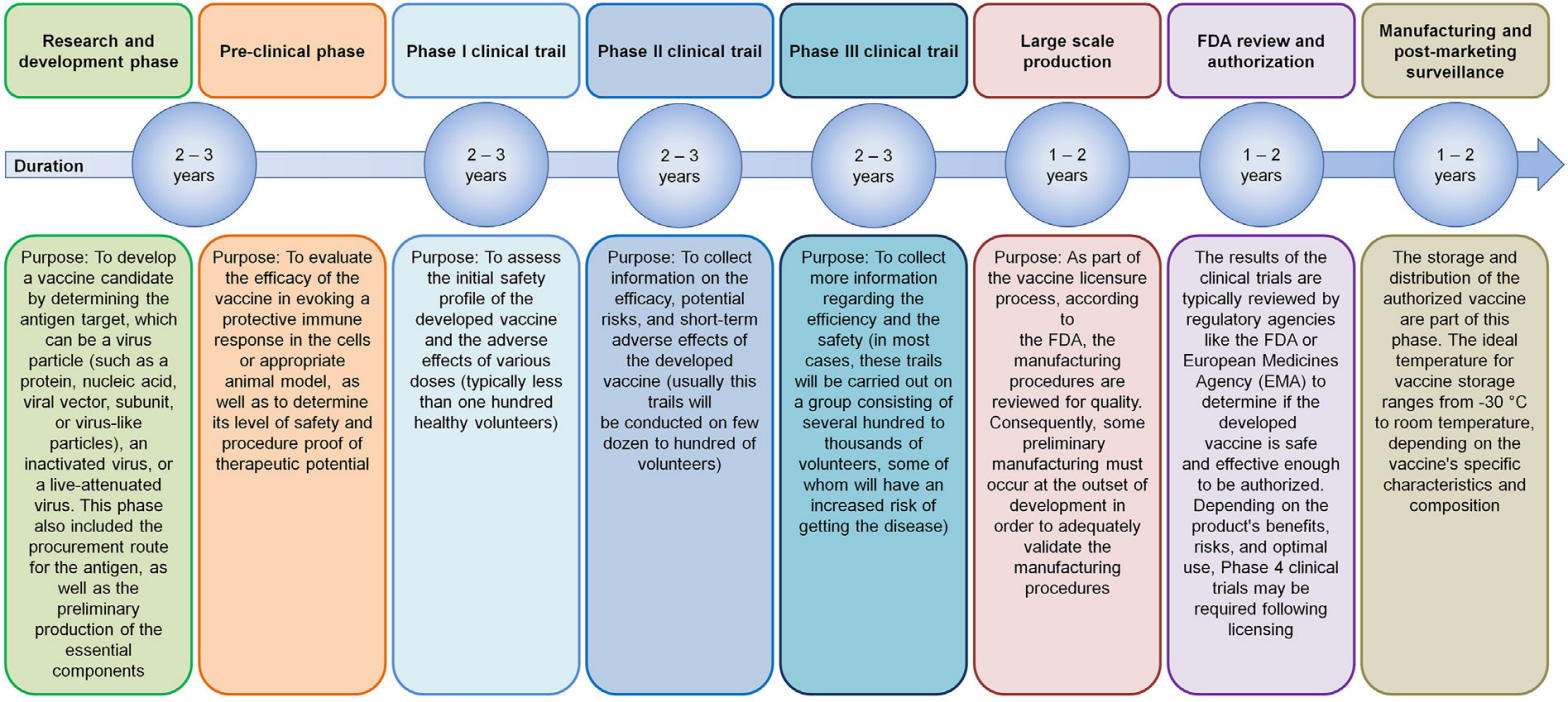
Conventional vaccine development process. The conventional process for developing vaccines includes several phases, beginning with research and development; then pre-clinical studies; then phase I trials in a relatively small number of control volunteers; and finally more extensive phase II and phase III trials. After that, the Food and Drug Administration or the European Medicines Agency evaluates the findings of the clinical trials to determine whether the vaccine is sufficiently effective and safe to be authorized and is also affordable. The final phase is manufacturing and post-marketing surveillance, which is completed after the vaccine has been made available for use by the general public and after it has been monitored for its overall effectiveness among the population. Any negative adverse effects that may arise from the vaccination programs are also documented in the vaccine reports in this phase.

**Figure 3:**
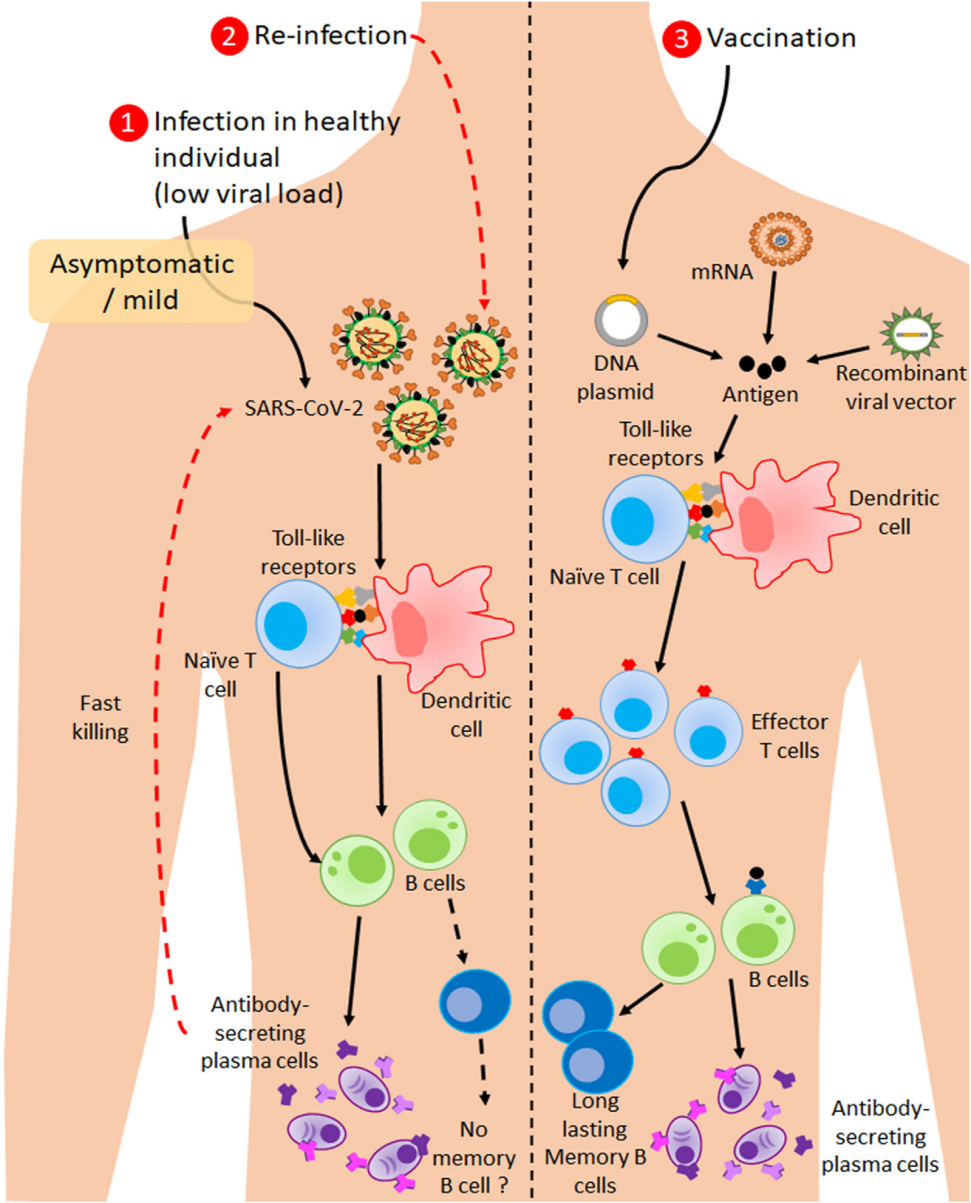
Schematic representation of the mechanisms of development of immunity to SARS-CoV-2 in three situations. 1) Infection in a healthy individual (low viral load). 2) Reinfection with SARS-CoV-2 when previously infected people did not have an adequate neutralizing IgG response, which formed only after their secondary infection. Usually, the innate immune response regulates the immediate host response to SARS-CoV-2 infection, which is then accompanied by an early antibody response. Development of high titers of IgM, IgA, and IgG virus-specific antibodies peaks 3–4 weeks after symptom onset, then decreases. In some cases, IgM and IgA levels exceed baseline levels 2 months after onset, whereas IgG levels remain elevated for as many as 3 months in most patients. Intriguingly, the frequency and durability of the COVID-19 antibody response tend to be correlated with the severity of the disease. In patients who experienced asymptomatic infection or mild illness, short-lived immunity has been noted, thus indicating that a low neutralizing antibody response has been established, with the virus effectively cleared by the innate and early antibody response. In asymptomatic/mild cases of COVID-19, the viral-clearing antibody response wanes very quickly (<90 days). Therefore, these people may be vulnerable to reinfection. High titers of NAbs have been found in the more extreme cases of COVID-19, and notably, no cases of reinfection of immunocompetent individuals have been identified. 3) Potential effects of different types of vaccines (DNA-based, RNA-based and non-replicating viral vector) after administration to the host. A vaccine works by imitating an infection caused by a particular pathogen for which the vaccine is developed. After the vaccine is injected into the host, the antigen inside the vaccine component is captured by antigen-presenting cells (APC), such as dendritic cells (DC), by phagocytosis. Subsequently, the DCs travel to a secondary lymphoid organ, such as lymph nodes, where immature T and B cells are situated. The antigen processed by the DCs is bound to the major histocompatibility complex (MHC) class II receptor on the DC surface. The antigen is then presented to immature T helper cells through binding the MHC class II to the T cell receptor (1st signal). Later, these T lymphocytes proliferate and mature. T helper cells activate B cells, which then proliferate into antibody-secreting plasma cells (2nd signal). These memory T and B cells are formed after the imitation infection by the vaccine ends.

Vaccines aid in developing immunity against particular pathogens by imitating an infection caused by the given pathogen. Although this type of imitated infection almost never causes illness, it stimulates the immune system to generate T cell populations and antibodies.^88^ As the body builds immunity, minor symptoms, such as fever, may develop and are considered normal. After the period of the imitated infection passes, the body is left with a population of memory T cells and B cells, which provide resistance against the particular disease in the future.^87^ Although the steps in the traditional method for developing new vaccines tend to be sequential and well established (Figure 2), some overlap exists between the stages. Because each phase is costly, particularly later phases, and every phase enhances understanding of whether the next step might be successful, the objective of this sequential strategy is partly to lessen the financial risk involved. The process can be halted at any point for a variety of reasons, including the detection of adverse events, such as major adverse effects, or if the data indicate that the vaccine is likely to be ineffective.^89^^,^^90^ Combining phases of vaccine development can accelerate the process in situations such as the upheaval created by the COVID-19 pandemic and the urgent need for a vaccine that is both effective and widely available worldwide.^91^ As of October 27, 2022, on the basis of the recent COVID-19 landscape report by the WHO, a total of 172 and 199 vaccine candidates are currently in clinical development and pre-clinical development worldwide, respectively.^92^ As shown in the same report, these vaccine candidates have been developed by using different platforms including, but not limited to, protein subunits (32.2%), RNA (23.4%), non-replicating viral vectors (13.5%), inactivated viruses (12.9%), DNA (9.4%), virus-like particles (3.5%) and replicating viral vectors (2.3%).^92^ Figure 3 summarizes the immunological defense mechanisms involved in developing immunity to SARS-CoV-2 in either infected or vaccinated individuals.^93^, ^94^, ^95^, ^96^, ^97^, ^98^, ^99^

### COVID-19 vaccine challenges

**Figure 4:**
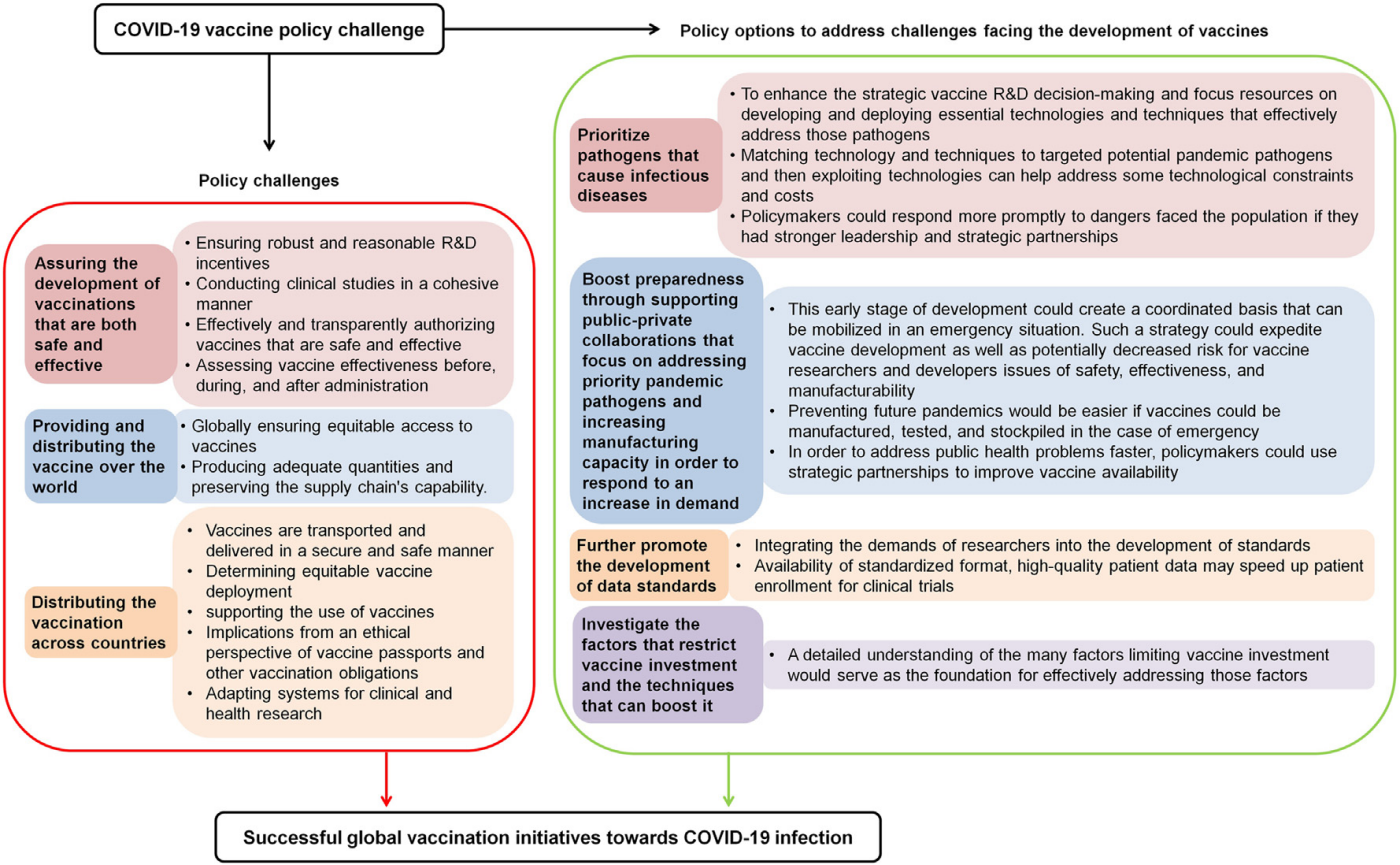
Challenges and policy options to address the challenges in the development of COVID-19 vaccines. The three primary “D” dimensions of obtaining broad global COVID-19 immunity by vaccinations are development, dissemination and deployment, which ensure continuing development of effective and safe vaccines, providing and distributing the vaccine globally, and deploying the vaccine across countries. To achieve these goals, 11 challenges must be addressed: maintaining robust and reasonable R&D incentives; conducting cohesive clinical trials; authorizing effective and safe vaccines; surveilling effectiveness after and during vaccination; ensuring equitable availability of the vaccine worldwide; producing adequate amounts and sustaining supply chain ability; safely transferring and supplying vaccines; deploying vaccines equitably; supporting vaccine use; addressing ethical concerns re: vaccine passports and other obligations; and adapting systems for clinical and health research. From the beginning of vaccine R&D until the adaption of the clinical system, financial and ethical decisions must be made.^102^ Because vaccine development methods and techniques face numerous challenges, the Government Accountability Office has outlined several policy options for overcoming these obstacles. Policymakers, including those in the US Congress, state and local governments, federal agencies, academic and research organizations, and private industry, could take one of several new actions as a result of these policy options.^89^

Infectious diseases can have enormous effects and devastating consequences on population health and country economies. Because prevention is always a better option than treatment, vaccines provide the best solution to combat infectious disease. Beyond providing long-term protection against infections, the ideal types of vaccines should be safe, easy to administer, simple and inexpensive to manufacture.^100^ Careful evaluations at each step are required to develop novel vaccines while ensuring effectiveness and safety. Hence, the pipeline for traditional vaccine development usually requires more than 10 years before reaching the general public.^101^ However, the highly infectious SARS-CoV-2 and the ensuing morbidity and mortality required urgent development of vaccines despite the challenges in vaccine development. The development of COVID-19 vaccines faces several policy challenges, as shown in Figure 4. Therefore, the Government Accountability Office in the USA has highlighted nine policy options that might help overcome challenges to vaccine development technologies and methods, as well as economic challenges (Figure 4).

#### Immune responses

Antigen selection for vaccine development requires a critical evaluation of whether to use whole-cell antigens (killed or weakened) or fragments of their viral materials (subunits). For the COVID-19 vaccine, vaccinologists are currently focusing on the S protein, which is believed to facilitate cell membrane fusion and viral entry.^103^ Of note, vaccination should trigger a proper robust immune response to ensure long-term protection while posing no harm to people. However, the emergence of several new strains and mutant variants has resulted in infections in fully vaccinated people and even reinfections among people who have recovered from COVID-19. Thus, to select the antigen with the optimal immune response, many studies are needed to fully understand how the immune system responds to SARS-CoV-2 ^55^. However, developing novel SARS-CoV-2 vaccines may be challenging because of the many unknowns regarding this virus. Therefore, further investigation is warranted.

#### Vaccine adjuvant and delivery system

In the early years of vaccine development, Louis Pasteur introduced inactivated pathogens (attenuated viruses) to stimulate the immune system.^104^ Since then, mainstream global vaccine development has evolved into a more minimalist design approach, wherein only one subunit of the peptide-based composition of the antigenic material is used in the formulation instead of the whole virus. Despite the higher safety and usually lower production cost for this type of vaccines, adjuvants are required because they are less immunogenic than inactivated whole-cell vaccines.^104^^,^^105^ Adjuvants are often designed to function as both immunostimulators and delivery systems to enhance vaccine immunogenicity. To date, aluminum salts, which are very inexpensive to produce, have been the most commonly used vaccine adjuvant. They have been approved by the Food and Drug Administration since the 1930s because of their safety and long-term protection.^106^^,^^107^ Despite showing an excellent safety profile and the longest history of use, aluminum adjuvant-containing vaccines also have some limitations. For example, aluminum-containing salts have been reported to induce strong humoral responses but not cellular responses.^105^ An ideal immunization for adaptive immunity must provide long-term protection that involves both humoral and cellular immunity for a rapid response and immunological memory to fight future infections by pathogenic agents.^87^ In addition, cellular immune responses have been suggested to be valuable for respiratory tract infections such as SARS-CoV-2, owing to the late onset of the production of antibodies after infection.^108^ In adaptive immunity, the CD8^+^ T cell subset is involved primarily in the cellular response, whereas CD4^+^ T cells are responsible for long-term protection against subsequent infection.^108^ Hence, the limited number of clinically approved adjuvants has restricted the choices for novel vaccines under development and prolonged the vaccine pipeline. The development of COVID-19 vaccines should, of course, meticulously consider every feature, including the type of adjuvants, to ensure optimal immunostimulation and antigen delivery. These considerations are complicated by the incomplete understanding of the exact mechanism of action of SARS-CoV-2, given its relatively recent emergence.

#### Financial commitment

The development of new vaccines such as those for COVID-19 is costly, particularly during this pandemic. As described, the typical timeline for previous vaccine development processes was 10 years or more. However, during the emerging pandemic disease, shortening of the timeline for the vaccine development was necessary. For instance, the mRNA-1273 vaccine candidate from Moderna entered a phase 1 clinical trial on March 16, 2020, less than 10 weeks after China had released the genetic sequence of SARS-CoV-2 ^55^. However, accelerating the process, such as by conducting research phases in parallel with other phases, poses a substantial financial risk, particularly in view of the many unknowns surrounding COVID-19. Nevertheless, the cost for the R&D of a new vaccine has not been disclosed by the pharmaceutical company, which has lacked transparency regarding the actual costs. However, on the basis of common costs for new drug discovery, the range for the true cost has been estimated to have been approximately $2.6 billion.^109^ Beyond the financial cost of R&D, advanced technologies, which are often available to only high-income nations, are required for the development of COVID-19 vaccines. Consequently, the development of novel COVID-19 vaccines is very challenging, particularly in low-and middle-income countries (LMICs), owing to the enormous financial commitments required for R&D.

Because of global inequality in terms of vaccine development and production capacity, programs must be developed to enable the distribution of vaccines on the basis of population needs rather than wealth or social status.^110^ Previously, operation Warp Speed, a US program, aimed to develop vaccines faster than ever before, to make them accessible for emergency use by the end of 2020 and to produce billions of doses by the end of the 2021.^91^ However, vaccine manufacturing and procurement cost billions of dollars in high-income nations, thus resulting in an uneven distribution of vaccines to LICs.^111^ Despite the recommendations made at the Global Vaccination Summit in favor of equal vaccine allocation, concerns remain that some governments might want to secure their citizens' vaccine supplies before sharing vaccines globally.^112^ Several countries, such as the United States and Europe, have indicated that vaccines will initially be delivered to their own citizens because of the high demand for vaccines. However, questions have been raised regarding the ethics of distributing resources unequally. Nonetheless, positive steps have been made, including by the COVAX initiative and AstraZeneca's announcement of a partnership with an Indian institute to provide appropriate doses to LMICs.^113^

The unequal distribution of vaccine supplies worldwide has substantially affected progress in vaccination in LICs. According to a United Nations report released on March 28, 2022, vaccination distribution has increased worldwide but still shows inequality. Only 1% of the 10 billion doses distributed worldwide went to LICs, thus leaving about 2.8 billion individuals waiting for their first dose.^114^^,^^115^ To date, slightly more than 3% of people have been vaccinated with at least one dose in LICs, as compared with 60% in high-income countries. As of the middle of 2022, the United Nations health agency sought vaccination for 70% of the world's population,^114^ a goal scarcely attainable. Approximately 39 high-income nations have already met the 70% target as of January 2022, whereas the Republic of Congo, Ethiopia, South Sudan and Nigeria still seek to acquire sufficient vaccines to vaccinate 10% of their populations. A total of US$19,87 billion in gross domestic product has been lost as a result of these four countries failing to meet the 40% target for vaccination by the year 2021.^116^

The COVAX initiative stands out as an international project designed to acquire and distribute vaccines in an equitable manner. However, it has not been able to procure sufficient doses to achieve its modest objective for 2021 of covering 20% of the LICS population.^111^^,^^117^ For example, Pfizer agreed to sell only 40 million doses to COVAX, and slightly more than 1 million doses had been delivered by mid-May 2021.^117^ By the end of May 2021, 34 countries worldwide had contributed doses, and many more had committed to the goals of COVAX.^118^ However, LMICs make up 6.5 billion of the world population, and high-income countries have purchased only 5.9 billion doses.^119^ As of January 12, 2022, 84 vaccines were in late developmental phases (phase II or III), and another 33 were in post-registration. Of the 28 vaccine manufacturers, Pfizer/BioNTech has made a public commitment to provide the most doses, exceeding 5.1 billion, followed by AstraZeneca, with approximately 3.6 billion doses.^120^ High-income countries have reserved most doses from Pfizer/BioNTech (83%) and Moderna (73%), but no bilateral agreements with low-income countries have been revealed. In contrast, AstraZeneca/Oxford, Johnson & Johnson and Novavax have had a larger distribution in LMICs. Vaccines produced in middle-income countries (India, Cuba, China and Russia) are restricted primarily to the country where the vaccine was developed or other middle-income countries. Meanwhile, LICs have conserved relatively smaller quantities of each of these vaccine candidates bilaterally.^120^ Therefore, AstraZeneca and the Serum Institute of India have collaborated to develop and supply vaccines to LMICs, in an attempt to partially avert this situation. Several organizations, such the WHO-led COVAX, African Vaccine Acquisition Task Team of African Union Africa in Africa,^121^ Serum Institute of India, Bharat Biotech, Premas Bio-tech and Zydus Cadila in India,^122^ have been established to work on vaccine development and procurement.^111^ In addition, donations could have assisted in vaccination of health professionals and vulnerable communities worldwide if they had been made more quickly and achieved more widespread distribution.^118^

This disparity between developed and the developing countries in terms of the prospects of achieving a timely solution for the COVID-19 pandemic echoes the commonly observed inverse relationship between income and tendency to contract the disease,^123^ given the pervasive outbreaks among low-income communities primarily working in manufacturing and the safety of online jobs available for the higher-income populace.^124^ Therefore, COVID-19 discourse is expected to increasingly pose important questions that address the principles of socioeconomic disparities related not only to the healthcare industry but also to broader aspects of our lives; it is also likely to provide a platform for recognizing, addressing and ameliorating the aforementioned disparities.

#### Vaccine hesitancy and adverse effects

The WHO defined vaccine hesitancy as a reluctance to be vaccinated despite the availability of vaccines.^125^ In 2019, the WHO designated hesitancy to be vaccinated as one of the ten threats affecting global health.^126^ Because the success of vaccination programs depends largely on the public's willingness to accept the vaccine, many studies have reported vaccine acceptance and hesitancy rates in various countries.^127^^,^^128^ Furthermore, several research groups have aimed to assess the determinants of, and factors driving, vaccine hesitancy and acceptance.^129^^,^^130^ Notably, many COVID-19 vaccine-associated studies have focused on healthcare workers (HCWs) in the early stages of the pandemic, because many countries prioritized vaccination of HCWs at the frontline of the pandemic response, who helped safeguard the lives of vulnerable populations.^131^

Although vaccine hesitancy and acceptance rates vary among populations,^132^^,^^133^ several studies from different countries have shown that vaccine hesitancy is associated with a central concern regarding the lack of trust in COVID-19 vaccine safety and efficacy, particularly given that the COVID-19 vaccines were developed at an unprecedentedly rapid pace.^132^^,^^134^^,^^135^ In addition, some psychosocial predictors, prior experience and preconceptions about vaccines, social media influence and trust in governments have been reported to be co-modulators of vaccine hesitancy/acceptance across different populations.^136^, ^137^, ^138^, ^139^ Moreover, since early 2021, multiple SARS-CoV-2 variants have emerged with reports of waning vaccine-induced immunity in various countries worldwide, thus leading to a surge in the demand for booster doses of COVID-19 vaccines. Studies have shown that booster immunization acceptance is attributable to the belief that boosters are necessary and efficient,^140^ as well as to altruistic reasons, such as family, patient and community health protection.^141^, ^142^, ^143^

In contrast, many post-authorization observational studies have been performed to assess the safety and effectiveness of COVID-19 vaccines. Independent studies not supported by vaccine development corporations have helped enhance public confidence in the safety of COVID-19 vaccines, thereby increasing vaccination rates in targeted populations.^144^ Generally, although post-vaccination adverse effects have been reported by most participants, studies have shown that serious safety issues of COVID-19 vaccines are extremely rare, and long-term adverse effects are unlikely.^145^, ^146^, ^147^ The most commonly reported post-vaccination adverse effects are fatigue, chills, dizziness, fever, headache, joint pain and myalgia.^148^, ^149^, ^150^, ^151^ In addition, post-vaccination adverse effects are mostly resolved within 3 days without medical intervention.^133^^,^^152^ However, the overall prevalence and severity of post-vaccination adverse effects may vary depending on the type of the vaccine.^133^^,^^144^^,^^153^^,^^154^

## COVID-19 therapeutics

The Milken Institute's COVID-19 treatment and vaccine tracker^155^ lists a total of 309 potential treatments from different categories, including repurposed drugs and drugs assessed in different stages of development against COVID-19, 195 of which have reached the clinical stage. In addition, five treatments (remdesivir, REGN-COV2, bamlanivimab, olumiant and chloroquine/hydroxychloroquine) have received emergency authorization. The chloroquine/hydroxychloroquine authorization was revoked thereafter, and 31 treatments have reached phase III trials.

This repurposing of available drugs has involved several types of antimalarial drugs (e.g., chloroquine), antimalarial and antibiotic combinations (e.g., hydroxychloroquine and azithromycin), antiviral drugs (e.g., nafamostat, camostat, bromhexine, favipiravir, remdesivir and lopinavir) and antihelminthic/antiparasitic agents (e.g., nitazoxanide and ivermectin), which have shown potential antiviral effects against SARS-CoV-2.^156^, ^157^, ^158^ Although prior studies did not identify specific antiviral drugs against SARS-CoV or MERS-CoV, reports have recently suggested that several available drugs may act as antiviral agents against SARS-CoV-2, particularly hydroxychloroquine, chloroquine and nafamostat.^159^^,^^160^ However, the clinical effectiveness of these drugs has not yet been fully evaluated, and several clinical trials remain underway.^160^

Furthermore, other types of repurposed drugs are expected to have regulatory effects on the immunity of patients with COVID-19, particularly those with severe disease. Anticoagulant treatments (e.g., heparin and nafamostat), for example, inhibit the cytokine storm and increase the percentage of lymphocytes.^159^^,^^161^^,^^162^ Some immune-based therapies (e.g., tocilizumab and interferon alpha-2B) are also expected to have similar effects but continue to await experimental evaluation.^156^ Other types of drugs have been repurposed and are currently being studied, such as antihypertensive drugs and non-steroidal anti-inflammatory drugs; however, no scientific evidence demonstrating the effectiveness of any drug or therapeutic compound against COVID-19 has been reported to date.

Another proposed therapeutic strategy for COVID-19 is using potent NAbs against SARS-CoV-2. Several studies have shown promising results supporting this strategy.^163^, ^164^, ^165^ One study has reported that two human monoclonal antibodies isolated from a convalescent patient with COVID-19 demonstrated potent SARS-CoV-2-specific neutralization activity *in vitro*. One of these antibodies showed inhibitory therapeutic and prophylactic activity against SARS-CoV-2 in rhesus monkeys.^163^ Structural studies of this antibody have indicated its ability to recognize an epitope that overlaps with ACE2-binding sites in the RBD of SARS-CoV-2 S protein.^163^ However, NAbs are not produced exclusively against the RBD of S protein regions. Another study has shown that 19 of 61 monoclonal antibodies isolated from five patients with COVID-19 potently neutralized authentic SARS-CoV-2 *in vitro*. Epitope mapping has indicated that half of these antibodies were directed against the RBD of S protein, whereas other antibodies were directed against the N-terminal domain of S protein^164^; therefore, both the RBD and N-terminal domain are immunogenic regions of S protein. However, further research remains needed before this therapeutic strategy can enter the clinical stage.

Combined and supplementary therapies have been suggested as potential therapeutic approaches. For example, an *in vitro* study has shown that using a combination of zinc ionophore (pyrithione) with Zn^2+^ potently inhibits SARS-CoV RNA replication in cultured Vero cells as well as SARS-CoV RNA-dependent RNA polymerase (RdRp) elongation.^166^ On the basis of the high sequence similarity between SARS-CoV and SARS-CoV-2 (79% at the genome level and 98% at the RdRp level), researchers have hypothesized that the antiviral effects of zinc or the aforementioned combination might be similarly effective against SARS-CoV-2.^167^ An interesting approach has suggested that combining zinc with chloroquine or hydroxychloroquine—which inhibit SARS-CoV-2 replication after an increase in the pH of the intracellular vesicles and also inhibit the intracellular virus particle delivery—might provide a promising treatment for COVID-19.^168^ Chloroquine or hydroxychloroquine may specifically target extracellular zinc and activate its entrance into the intracellular lysosomes, where it interferes with SARS-CoV-2 RNA replication and RdRp elongation activity, thereby enhancing the efficacy of these repurposed drugs. Furthermore, zinc, vitamin D, vitamin C and N-acetylcysteine have been suggested to have a potential role in the prevention and treatment of COVID-19. However, clinical evidence supporting the use of those supplements for these purposes is insufficient.^169^^,^^170^ Taking over-the-counter doses of these supplements in the absence of a confirmed nutritional deficiency might pose risks to the treatment outcomes.^169^ Thus, further research is required before doses above those recommended for these supplements could be approved.

**Figure 5:**
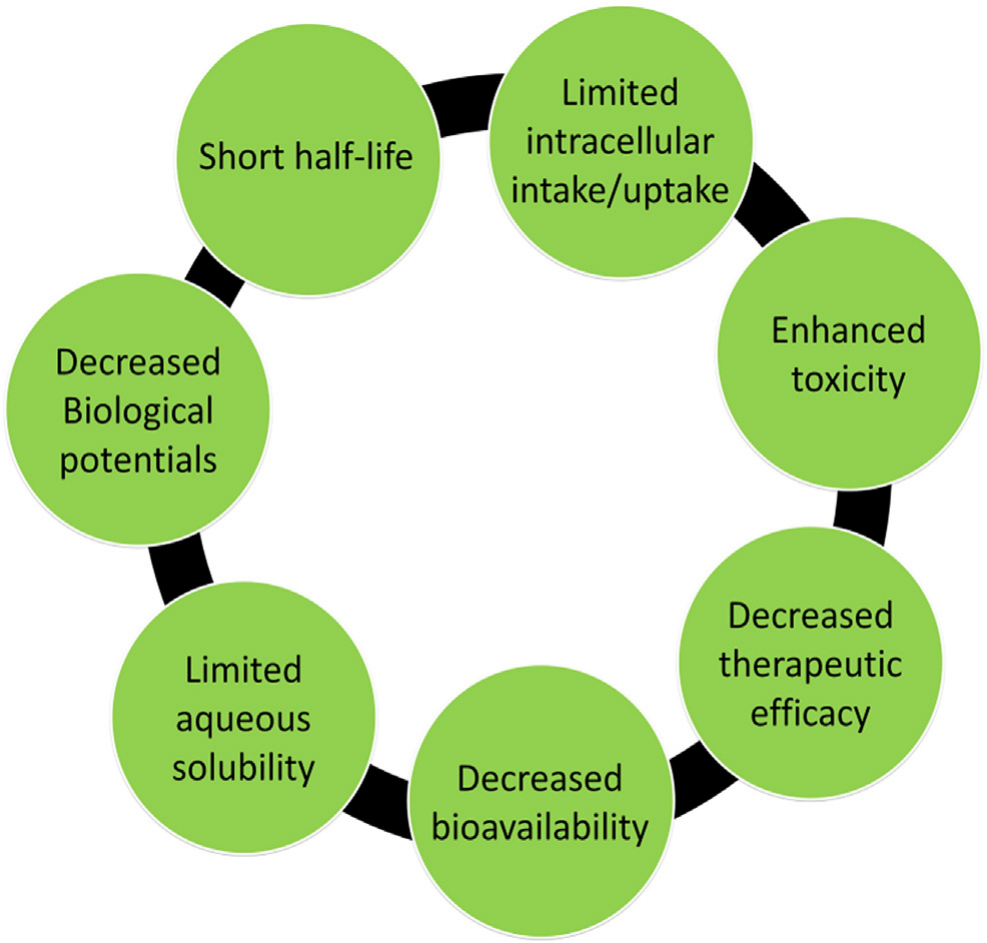
Challenges regarding repurposed drugs against COVID-19.

Overall, massive efforts have been made worldwide toward the development of COVID-19 therapeutics, and many clinical trials have aimed at evaluating the effectiveness of repurposed drugs. Given that their chances of reaching very advanced phases are moderate to high, we summarize the potential challenges of repurposed drugs^171^ in Figure 5.

## Convalescent plasma therapy for COVID-19

According to the WHO, most studies have shown that people who have recovered from COVID-19 have antibodies against the virus; however, some of those people have very low levels of NAbs in their blood.^6^^,^^172^ CP is a convalescent blood product obtained from patients who have recovered from a viral infection and have developed humoral immunity against a particular pathogen.^173^ The isolated plasma from the whole blood of recovered patient donors has a high concentration of NAbs against the virus. Hence, the administration of CP to patients with severe SARS-CoV-2 infection might possibly decrease viral replication and disease severity via specific antigen–antibody binding.

In general, CP is a passive antibody therapy that depends on the transfer of pathogen-specific antibodies from a recovered patient to aid in the prevention or treatment of disease.^5^ Antibody treatment can also be used to treat individuals already exhibiting symptoms with varying severity. In contrast, passive antibody therapy is most successful when applied prophylactically, or when used immediately after the onset of signs and symptoms.^174^ Passive antibody administration through transfusion of CP may be the only short-term method available for providing direct immunity to susceptible patients in a timely manner. Numerous cases have been reported in which CP has been used successfully for treatment and/or postexposure prophylaxis against infectious diseases, including in prior coronavirus outbreaks (i.e., SARS-CoV-1 and MERS-CoV).^175^

The development and implementation of CP therapy is less complicated than that of vaccines and monoclonal antibodies. In addition, CP can adapt to a variety of conditions. The only requirements are the availability of disease survivors who can donate plasma and a standard blood collection infrastructure to collect and distribute the CP.^175^ Individuals who are eligible to donate CP must have a history of COVID-19, as confirmed by approved molecular testing (e.g., nasopharyngeal swab) or the presence of antibodies against SARS-CoV-2. In addition, symptoms including fever, cough and shortness of breath must have resolved at least 14 days before donation of CP.^175^ In light of the emergence of variant SARS-CoV-2 strains, CP donated by survivors of infections with variant strains is an immediately deployable treatment option for patients who have been identified as having a variant infection, whereas other immune treatments may require (re)development of more specific mechanisms to target new viral strains.^176^, ^177^, ^178^

Three essential principles of passive antibody therapy must be met by CP. To be effective against SARS-CoV-2 infection: i) CP must contain specific antibodies against the SARS-CoV-2 virus; ii) a sufficient quantity of anti-SARS-CoV-2 antibodies must be produced in the plasma to improve the outcome of the disease; iii) for optimal benefits, the plasma must be administered early after the onset of symptoms.^179^

CP therapy was first recognized as a potential antiviral therapy during the Spanish influenza pandemic approximately 100 years ago.^180^ CP administration substantially decreases mortality among patients with severe acute respiratory infections, by 75%.^181^ Beyond SARS-CoV, CP therapy has been successful in the treatment of MERS-CoV-2 and H1N1 viral infections.^182^ Despite the effectiveness of the CP therapy against previous viral infections, this type of therapy was unable to improve the survival rate during an Ebola virus outbreak (141). Because SARS-CoV-2 shares similar genomic sequences with SARS-CoV and MERS-CoV, CP therapy can be considered a promising treatment strategy with the potential to help the infected patients develop immediate short-term passive immunization before the availability of a new generation of COVID-19 vaccines. In addition, owing to the rise of different strains of the COVID-19 pathogen arising from genetic modifications with consequences in mutability, CP therapy offers an advantage in providing artificially acquired, passive immunity against the local infections in communities.^173^ Recent studies on CP therapy for COVID-19 have reported that CP with high levels of NAb titers from recovered patients with COVID-19 ameliorates clinical symptoms by decreasing viral load, while posing low risk of treatment^183^ and no significant transfusion-associated morbidity or mortality.^184^ A recent study has found that 15% of 2341 enrolled men and women were less likely to die from COVID-19 within 1 month after receiving an injection of CP than patients who received an inactive saline placebo or did not receive the CP therapy.^185^

Moreover, a meta-analysis of international randomized clinical trials (RCTs) among hospitalized, non-critically ill patients with COVID-19 has been conducted to evaluate the effects of CP on patients with COVID-19. The findings were derived from eight recently completed studies conducted in Belgium, the United States, India, Brazil, Spain and the Netherlands. The COVID-19 CP was not consistently associated with either benefits or harms in therapy.^186^ In addition, CP was not found to have an effect on clinical outcomes for typical patients.^186^ However, patients with type A and AB blood groups, and patients with preexisting diseases such as diabetes or cardiovascular disease were most likely to benefit from the CP therapy.^186^ However, another study has reported the benefits of CP therapy for older outpatients within the first 72 h of disease onset.^187^ Monoclonal antibody-based therapy has been shown to be helpful, but only for outpatients who have recently been diagnosed with SARS-CoV-2 infection.^188^^,^^189^ As a consequence, the lack of a clear benefit of CP even in patients with recent symptom onset has been rather unexpected, thus implying that the window of opportunity for antibody-based therapy may be limited and associated with the stage of illness rather than with symptom onset time.^186^

Furthermore, the Continuous Monitoring of Pooled International Trials of Convalescent Plasma (COMPILE) for COVID-19 Hospitalized Patients study has established and validated a CP treatment benefit index.^190^ COMPILE included a total of 2369 hospitalized adult patients (18 years of age or older) who had COVID-19 and who might or might not have required oxygen supplementation, but did not require mechanical ventilation. The treatment benefit index score represents the estimated relative advantage that a patient is expected to gain from CP treatment compared with a treatment without CP.^190^ Another recent CT study has been conducted on 941 patients who were hospitalized with COVID-19 for 3 or fewer days and required noninvasive oxygen supplementation.^191^ In this trial, patients receiving large doses of CP who were not taking any other drugs, such as remdesivir or corticosteroids, were found to be likely to benefit from CP treatment.^191^ These preliminary findings have reinforced the idea that CP may serve as a viable therapeutic option, particularly when alternative medicines are not yet available. However, the lack of an overall advantage from CP therapy in this trial,^191^ as well as other trials on CP^192^, ^193^, ^194^ in hospitalized patients with severe to life-threatening disease, suggests that patients with less severe disease and older patients may benefit most from CP treatment. Moreover, CP obtained from donors who had been previously infected and vaccinated afterward has been found to possess antibodies with sufficient quantities and diversity to provide further protection against new COVID-19 strains.^191^ Genetic mutations are typical of viral evolution during the course of the COVID-19 pandemic. Consequently CP has the potential to provide a successful treatment option that keeps pace with the mutations better than synthetic treatment options, such as monoclonal antibody therapies, which tend to become less effective over time because of mutations and must undergo redevelopment to address the new variants.^185^

Several key challenges in developing CP therapy that must be considered. First, plasma transfusions are associated with potential risks of adverse effects. Previously, a patient with Ebola virus disease had developed acute respiratory distress syndrome after plasma transfusion from the recovered patients, possibly because of transfusion-associated acute lung injury.^195^ A recent case study on CP for COVID-19 has also reported that a patient developed erythema, hypoxia and hypotensivity with increased dyspnea within 10 min of plasma transfusion.^196^ Limitations also exist regarding the risk of transfusion-transmitted diseases.^197^ Table 2 shows the characteristics of recent RCTs evaluating CP therapy for SARS-CoV-2 infection.

**Table 2:** Characteristics of RCTs evaluating convalescent plasma therapy for SARS-CoV-2 infection.

Trial registration number	Study phase	Year	Location	Status	Number of enrolled patients	Patient setting	Primary outcome measure	Mortality rate, planned mortality time point	Plasma titer, assay	References
NCT04385199	3	2021	Italy	Completed	182	Inpatients with supplemental oxygen	Proportion of patients without progression in severity of pulmonary disease, defined as worsening of 2 points in the ordinal scale of WHO within 14 days	16.67%, 28 days	Low titer: ≥1:200 S-RBD IgG	^198^
CTRI/2020/04/024775	2	2020	India	Completed	464	Inpatients with supplemental oxygen	1. Progression to severe ARDS (P/F ratio 100) or 2. All-cause mortality at 28 days	14.01%, 28 days	No minimum cut-off described	^199^
NCT04356534	N/A	2021	Bahrain	Completed	40	Inpatients with supplemental oxygen	Requirement for invasive ventilation	7.5%, 28 days	No minimum cut-off described	^200^
NCT04345523	2	2021	Spain	Completed	350	Inpatients with or without supplemental oxygen	Category changes in the “7-Ordinal Scale” ranging from 1 (not hospitalized, no limitations on activities) to 7 (death)	4.9%, 29 days	Confirmed High-titer: >1:80, neutralizing assay	^201^
NCT04346446	2	2020	India	Completed	29	13.7%, 28 days	Proportion of patients remaining free of mechanical ventilation	Inpatients with supplemental oxygen	No minimum cut-off described	^202^
CTRI/2020/05/025209	2	2021	India	Completed	80	Inpatients with supplemental oxygen	All-cause mortality on day 30 after enrollment and identification of immunological correlates of response to CP	30%, 30 days	No minimum cut-off described	^203^
NCT04381858	3	2020	Mexico	Completed	196	Inpatients with supplemental oxygen	1. Mean hospitalization time 2. Mean oxygenation index evolution 3. Rate of severe acute respiratory distress syndrome (ARDS) 4. Rate and time to death 5. Mean time with invasive mechanical ventilation	45.2%, 28 days	No minimum cut-off described	^204^
NCT04433910	2	2021	Germany	Completed	106	ICU	Composite endpoint of survival and no longer meeting criteria for severe COVID-19	14.3%, 21.0%, and 26.7%, 21, 35 and 60 days, respectively	Confirmed high-titer: ≥1:20 neutralizing antibodies	^205^
NCT04359810	2	2020	USA and Brazil	Completed	223	Inpatients with or without supplemental oxygen	Day 28 severity outcome	28 days	Low titer: ≥1:400 S-RBD IgG, quantitative ELISA	^206^
NCT04441424	N/A	2020	Iraq	Completed	49	Inpatients with supplemental oxygen	Death versus survival of treated patients	6.1%, Up to 8 weeks	No minimum cut-off described	^207^
NCT04383535	N/A	2020	Argentina	Completed	333	Inpatients with or without supplemental oxygen	Ordinal outcome with six mutually exclusive categories describing the patient's clinical status during follow-up (ranging from 1 (death) to 6 (discharge))	11.1%, 30 days	Confirmed high-titer: ≥1:800 S-RBD IgG, COV-IDAR IgG test	^208^
NCT04392414	2	2020	Russia	Completed	60	Inpatients with or without supplemental oxygen	Number and proportion of patients with normal body temperature (≤37.2 °C) at day 1, 2, 3, 4, 5, 6, 7 after the start of therapy	6.06%, 30 days	Confirmed high-titer: ≥1:1000 S-RBD IgG, in-house assay	^209^,^210^
NCT04332835	2–3	2020	Colombia	Completed	92	Inpatients with supplemental oxygen	1. Change in viral load 2. Change in immunoglobulin G COVID-19 titers	8.0%, 7, 14 and 28 days	Confirmed high-titer: IgG ≥ 1/3200 and IgA ≥ 1/800 by EUROIMMUN; all transfused plasma presented neutralizing antibodies ≥1/256	^209^,^211^
NCT04366245	2	2020	Spain	Completed	72	Inpatients with supplemental oxygen	1. Incidence of adverse events and serious adverse events, grade 3 and 4, associated with the product under investigation or the administration procedure, graduated according to CTCAE. 2. Death from any cause 3. Need for mechanical ventilation 4. SOFA scale ≥3 at 72 h after randomization or an increase in 2 points or more from the basal level	2.44%, 14 and 28 days	Low titer: Vircell SL (Spain) test, correlates to ≥1:320 S-RBD IgG	^209^,^212^
NCT04362176	3	2021	USA	Completed	1000	Inpatients with or without supplemental oxygen	COVID-19 7-point Ordinal Clinical Progression Outcomes Scale (ranging from 1 (not hospitalized with resumption of normal activities) to 7 (death))	Not defined, 14 and 28 days	50% neutralization titer >1:50 (neutralizing antibodies) are selected for transfusion in the trial, Abbott™ ARCHITECT™ platform and the RBD Luminex assay	^213^
NCT04323800	2	2021	USA	Completed	180	Outpatients	1. Efficacy of treatment at day 28 2. Safety of treatment with high-titer anti- SARS-CoV-2 plasma versus control-1 and control-2	0%, 28 days	Confirmed high-titer: ≥1:320 neutralizing antibodies, Euroimmun ELISA	^214^
NCT04364737	2	2021	USA	Active, not recruiting	941	Inpatients with supplemental oxygen	The participant scores on the 11-point WHO Ordinal Scale for Clinical Improvement on day 14 after randomization (range from 0 to 10, with 0 indicating uninfected and no viral RNA detected and 10 indicating death)	4.0%, 14 and 28 days	Confirmed high-titer: ≥1:100 neutralizing antibodies, ELISA	^215^
NCT04373460	2	2020	USA	Active, not recruiting	1225	Inpatients with or without supplemental oxygen	1. Cumulative incidence of hospitalization or death before hospitalization 2. Cumulative incidence of treatment-associated serious adverse events 3. Cumulative incidence of treatment-associated grade 3 or higher adverse events	0.2%, 28 days	Confirmed high-titer: 1:320 SARS-CoV-2 spike protein titers, Euroimmun ELISA	^16^,^216^
IRCT20200310046736N1	2–3	2020	Iran	Not yet recruiting	30	Outpatients	Improvement in the levels of cytokine storm indices	26.7%, 7–14 days	No minimum cut-off described	^217^
NCT04338360	N/A	2020	USA	Approved for marketing	3082	Inpatients with or without supplemental oxygen	Death within 30 days after plasma transfusion	26.9%, 30 days	Confirmed high-titer: ≥1:160 neutralizing antibodies	^192^
NCT04381936	3	2021	United Kingdom	Recruiting	11558	Inpatients with or without supplemental oxygen	All-cause mortality	24.3%, 28 days	Confirmed high-titer: ≥1:100 neutralizing antibodies	^218^
NCT02735707	3	2021	Worldwide	Recruiting	2011	ICU	1. All-cause mortality 2. Primary endpoint for patients with suspected or demonstrated SARS-CoV-2 infection	37.2%, 90 days	Confirmed high-titer: ≥1:100 neutralizing or equivalent	^219^,^220^
NCT04873414	3	2021	Indonesia	Recruiting	364	Inpatients with supplemental oxygen	Mortality of patients with COVID-19 treated with CP (number of deaths from the initiation of CP treatment until hospital discharge or death)	Not defined, 82 days	No minimum cut-off described	^221^
NCT04344535	2	2021	USA	Terminated	82	Inpatients with and without supplemental oxygen	1. Number of days of invasive mechanical ventilation through 28 days post randomization 2. Patients who died during this time period were assigned 0 ventilator free days	16.1%, 90 days	Confirmed high-titer: >1: 320, neutralizing assay	^222^
NCT04342182	3	2020	Netherlands	Terminated	86	Inpatients with or without supplemental oxygen	Comparison of mortality in the 300 ml CP group and the control arm	19.8%, 60 days	Confirmed high-titer: >1:80, neutralizing assay	^223^
ChiCTR2000029757	N/A	2020	China	Terminated	103	Inpatients with supplemental oxygen	Patient discharged alive or decrease of 2 points on a 6-point disease severity scale (ranging from 1 (discharge) to 6 (death))	19.4%, 28 days	Confirmed high-titer: ≥1:640 S-RBD IgG, in-house assay	^224^
NCT04479163	N/A	2020	Argentina	Terminated	160	Outpatients	Development of severe respiratory disease defined as a respiratory rate >30 and/or an O_2_ saturation <93%	3.8%, 25 days	Confirmed high-titer: >1:1000 against SARS-CoV-2 spike protein (COVIDAR IgG)	^225^
NCT04348656	3	2021	USA	Terminated	940	Inpatients with supplemental oxygen	Endpoint of the need for intubation or patient death	30.3%, 30 days	Confirmed high-titer: ≥1:100 neutralizing antibodies	^178^

ELISA, enzyme linked immunosorbent assay; N/A, not applicable; SOFA, sequential organ failure assessment; CTCAE, common toxicity criteria scale.

## Herd immunity and COVID-19

When a population is immune to an infectious disease, either through vaccination or immunity acquired through earlier infection, this is referred to as herd immunity or population immunity.^6^ Herd immunity can offer indirect protection against the contraction of infectious disease, particularly for the most vulnerable people, such as those with comorbid conditions, as well as infants and older people. Before vaccination, herd immunity could be achieved only by exposing a very large number of people in the community to the disease in hopes that they would develop protective antibodies.^226^ The phenomenon of herd immunity, which occurs naturally, was first observed in the 1930s, after many adolescents had developed immunity to measles.^227^ Herd immunity is an important concept for understanding the short- and long-term effects of vaccination programs, and many existing examples illustrate this concept. In one of the most prominent examples, epidemics of common pediatric diseases, such as mumps, pertussis, polio, rubella, measles and chickenpox, occur at regular intervals.^228^^,^^229^ In addition, an indirect herd protection has been documented after the introduction of conjugate vaccines against pneumococcal and Haemophilus infections.^230^ Herd immunity is supported by existing examples and historical findings, and has again become the most hotly debated topic in science in the 21st century, in the midst of the SARS-CoV-2 pandemic. Concerns have been raised regarding the effectiveness of herd immunity in minimizing SARS-CoV-2 infection rates among susceptible populations.^231^

The basic reproduction number, or R_0_ (pronounced “R naught”), is the main parameter of the herd immunity threshold. It describes the average number of secondary infections acquired by the introduction of a single infectious individual into a fully susceptible population.^232^ If the population is not immune to the infection, then an infected person can theoretically infect as many as four more people throughout the infectious period if the R_0_ is 4. For example, if R_0_ is 4, the herd immunity threshold is 0.75, which can be calculated with the formula 1–1/R_0_.^233^ The interaction between the host and pathogen determines R_0_ in a given population, and this value may vary among pathogens. Variables such as population characteristics and the dynamics of disease transmission may affect R_0_.^234^

R_0_ is widely used in the research of infectious disease dynamics, as one of the most fundamental metrics.^235^, ^236^, ^237^ In most cases, the R_0_ for an infectious disease event is given as a single number or low-to-high range, and its interpretation is as follows: if R_0_ > 1, the outbreak is predicted to continue, and if R_0_ < 1, it is expected to cease.^232^ R_0_ can be used to estimate the percentage of a population that must be vaccinated to eliminate an infection; hence, it is commonly used to estimate the potential size of an outbreak or epidemic.^234^ Many communicable diseases have reported R_0_ values, including Ebola virus disease, measles, influenza, polio, acquired immunodeficiency syndrome (AIDS) and a variety of vector borne infectious diseases.^238^, ^239^, ^240^

The threshold of herd immunity to alleviate or slow the COVID-19 pandemic depends on the R_0_ for SARS-CoV-2. This threshold is described as the proportion of a population immune to an infectious disease, be it as the result of vaccination, natural infection or innate immunity, such that serial transmission of the infectious agent of the communicable disease is precluded or substantially decreased.^226^ However, because COVID-19 remains actively spreading, the precise R_0_ number is difficult to determine. Therefore, over time, the value of R_0_ is inconstant, varying over a wide range of values at different areas in the interconnected world, depending on the outbreak stages and policymakers' strategies to control the infection, which differ across countries and often across regions within a single country.

Initial R_0_ predictions for COVID-19 differed among different populations. Most values have ranged from 1.66 to 3.58.^1^^,^^241^, ^242^, ^243^ For instance, the first R_0_ value was reported in Wuhan, China, to be 2.2.^244^ More recently, however, the team from the University of Oxford has estimated the R_0_ to be 2.63.^245^ Variants including Alpha, Gamma and Beta have reproductive numbers estimated at 4.7–4.9,^246^ whereas the Delta variant has a reproductive number of approximately 5.^247^^,^^248^ Preliminary estimates of reproductive numbers of the Omicron variant are 4.2 times greater than those of the Delta variant.^249^ Thus, as R_0_ values increase, estimates of the herd immunity threshold also increase. R_0_ is influenced by demographic and social factors, including individual behavior, public health indicators, population density and cultural attitudes, thus leading to wide variations in herd immunity threshold estimations. In addition, the herd immunity threshold formula is dependent on the notion that COVID-19 has the ability to mutate to escape the immune system, and also on the nature of immune responses toward non-systemic respiratory viruses in general, which appear to be transient and only partially effective.^250^^,^^251^ Thus, the central epidemiological question is what proportion of the population should be infected for herd immunity to be achieved and the community protected from contracting and/or spreading infectious SARS-CoV-2. However, given the relatively high number of people who should be infected and might potentially die from SARS-CoV-2 infection for herd immunity to be achieved, strategies to achieve the acquired immunity via natural COVID-19 infection would be difficult to accomplish. Therefore, the WHO recommends that herd immunity against COVID-19 be obtained through vaccination rather than by exposing people to the virus that causes the disease, to avoid unwanted new cases and increased mortality.^6^

In 2016, the annual number of deaths due to measles was less than 100,000, a number significantly lower than the more than 2 million deaths due to COVID-19 reported during the pre-vaccination period.^252^ To achieve herd immunity, the WHO has set the limit of 95% of the population that should be immunized, because of the high infectivity of measles.^253^ Accordingly, despite the lower mortality rate of COVID-19, its high infectivity requires a high proportion of the population to develop immunity to stop the spread of the infection, thereby reflecting the challenges in creating herd immunity. Deciding on priority groups to receive COVID-19 vaccination is challenging not only because of the limited supply of vaccines with early approval for HCWs, older people, and the middle-aged community,^254^ but also because of direct effects on herd immunity. Another issue is the duration of immunity offered by the vaccination program. Because SARS-CoV-2 remains relatively new, the duration of the immunity memory, particularly in terms of epitope stability due to viral mutation, remains a large unknown.^255^

Both vaccinated people and convalescent patients with COVID-19 have shown lower binding of NAbs,^256^, ^257^, ^258^ and vaccine effectiveness has been lower in regions where the Gamma and Beta strains are more widespread.^259^^,^^260^ In addition, Omicron has 32 S protein mutations that decrease NAb titers by 27–127 times relative to that of wild-type COVID-19.^261^ The Omicron variant of the coronavirus, which is spreading more quickly than previous variants, is unlikely to help countries achieve herd immunity against COVID-19, wherein individuals would become immune to the virus with decreased spreading rates.^262^ The Omicron variant's transmissibility is enhanced because it is more effective than its predecessors at infecting people who have been vaccinated or have previously been infected. Growing evidence indicates that the coronavirus will continue to develop new ways to overcome immune responses.^262^ According to recently published studies, vaccination effectiveness against Omicron-associated symptoms is lower than that for other variants, and it decreases over time. Consequently, more people who have been vaccinated against Omicron are at risk of developing severe disease.^263^, ^264^, ^265^

However, these studies also suggest that vaccination continues to provide substantial protection against severe disease and hospitalization, owing to the variety of diseases caused by the Omicron variant. People who have received a booster dose may be more protected than those who have received only the initial course, as suggested by the most recent evidence, including real-world effectiveness data. In South Africa, individuals who have received two doses of the Pfizer–BioNTech vaccine, for example, have been found to have as much as 70% protection against hospitalization.^264^ Additionally, during the proxy Omicron period, the BNT162b2 vaccine maintained its effectiveness, albeit at a lower level, against hospital admission due to COVID-19 thought to have been caused by the Omicron variant than the Delta variant. The UK surveillance report has also revealed that after a booster dose of either the Pfizer or Moderna vaccine, the vaccination effectiveness against symptomatic disease among Omicron-infected patients was 60%–75% in the first 2–4 weeks, after which it decreased to 25%–40% in the next 15+ weeks.^265^

Despite the high infection rates, the two-dose COVID-19 vaccine strategy has significantly decreased hospitalizations and deaths.^266^^,^^267^ Similarly to other vaccines, the second dose loses some of its protective efficacy within several months.^268^^,^^269^ Previous studies have shown that, as any with other vaccine, COVID-19 vaccines show a decline in the levels of binding and NAbs with time.^268^, ^269^, ^270^ A preprint has indicated that at least 20 weeks after the second dose of the SARS-CoV-2 vaccine, the efficiency of ChAdOx1 nCoV-19 (Oxford-AstraZeneca) was 44.1%, and that of BNT162b2 (Pfizer-BioNtech) was 62.5%.^271^^,^^272^ In Qatar, researchers have observed similar findings, with the exception that the effectiveness against hospitalization and death remained strong even after 6 months.^273^, ^274^, ^275^ Similarly, a large study conducted in the USA has found that the risk of infection significantly increases 6 months after vaccination, and mRNA-based vaccines have a substantially lower increase in risk than the viral vector-based vaccine Ad.26.COV2.S (Janssen).^276^ Booster doses of BNT162b2 after vaccination have been shown in two Israeli studies to increase protection against symptomatic infection by 93.1%.^277^^,^^278^ Both humoral and cellular responses to SARS-CoV-2 have been reported to be enhanced by booster doses in the COV-BOOST randomized controlled trial in the UK, with adverse effects similar to those seen after initial vaccination.^279^ Moreover, the effectiveness against infection for both mRNA (mRNA-1273 [Moderna] and BNT162b2) and viral vector (ChAdOx1 nCov-19) COVID-19 vaccines has been shown to dramatically decline over 5–8 months, with respect to that 1 month after the second dose.^280^ Moreover, a booster dose administered 6 months after the second vaccination has been found to restore the vaccine's efficacy to levels greater than those reported 1 month after the second vaccination. Those who received a heterologous booster schedule experienced a higher rate of systemic reactogenicity after vaccination than those who received a homologous booster, although both groups experienced very mild adverse effects after the booster vaccination.^280^ Moreover, according to a study conducted in Canada, the effectiveness of vaccines in vaccinated individuals (compared with unvaccinated individuals) increased with each additional dose, and provided 49% (95% confidence interval 43%–54%) protection against infection, 69% (61%–76%) protection against symptomatic infection and 86% (81%–90%) protection against severe outcomes after the fourth dose.^281^ During the Omicron dominant era, results have suggested that a fourth dose of the mRNA COVID-19 vaccine (mRNA-1273) provides better protection against infection, symptomatic infection and severe outcomes for people living in long-term care centers.^281^ According to the available findings, vaccine boosters are safe for the general public and effective at restoring immunity after a period of waning protection. Homologous booster schedules have more favorable safety profiles than heterologous booster schedules.

The efficacy of COVID-19 vaccines against asymptomatic infection may still be associated with transmission and is generally less efficient and may be less adequately estimated than that against symptomatic infection.^282^^,^^283^ In a retrospective study conducted on HCWs who received the Pfizer vaccine, the vaccine efficiency for avoiding symptomatic infection was 97%, but was only 86% for avoiding asymptomatic disease.^284^ The efficacy of the Moderna vaccine against asymptomatic disease was only 63%, as compared with 93.2% against symptomatic infection, whereas the efficacy of the AstraZeneca vaccine with the original dose regimen was only 3.8%.^260^^,^^285^ Therefore, “universal vaccinations” as vaccine regimens that generate longer-lasting immunity and cover a wider range of potential future mutations may help decrease some of the problems associated with herd immunity against respiratory viruses. However, this type of vaccine remains under investigation.^286^

Herd immunity against COVID-19 can be safely achieved only by vaccinating large numbers of people, thereby decreasing overall viral transmission in the entire population. On the basis of the opinion of many experts, increasing evidence suggests that 80%–90% of the population would be required to have COVID-19 immunity for herd immunity to be effective.^287^ Vaccinations and prior infection will help enhance herd immunity against COVID-19, thus decreasing the severity of the disease for people who are infected or reinfected.^262^ However, to determine vaccine effectiveness against various outcomes (such as severe disease and subsequent transmission), vaccine effectiveness in various population subgroups and against various variants, as well as vaccine duration of protection, real-world vaccine effectiveness studies after implementation are required.^265^ Herd immunity threshold estimates must therefore consider not only partial vaccine effectiveness, but also changes in critical parameters associated with mutations and with dynamic and steadily declining immunity. Table 3 shows recent RCTs that have indicated assessment of efficacy of candidate vaccines against SARS-CoV-2 infection.

**Table 3:** Assessment of efficacy of different vaccines against SARS-CoV-2 infection among RCTs (as of October 27, 2022).

Vaccine candidate	Developers	Doses	Trial phase	Clinical trial numbers	Efficacy of vaccine against hospitalization for severe and non-severe SARS-CoV-2 infection	Efficacy of vaccine against emergency department visits associated with SARS-CoV-2 infection	Efficacy of vaccine against severe and non-severe SARS-CoV-2 infection	Seroconversion rates (efficacy)	Immunogenicity and safety of a booster dose
CoronaVac; inactivated SARS-CoV-2 vaccine (Vero cells)	Sinovac Research and Development Co., Ltd	2	Phase 4	NCT04756830			✓	✓	✓
				NCT04789356			✓	✓	✓
				NCT04801888	✓		✓	✓	✓
				NCT04894227				✓	✓
				NCT04892459	✓		✓	✓	✓
				NCT04953325	✓		✓	✓	✓
				NCT04962308				✓	✓
				NCT05057169				✓	✓
				NCT05148949	✓		✓		✓
				NCT05112913				✓	✓
				NCT05165966					✓
				TCTR20210308003					✓
Inactivated SARS-CoV-2 vaccine (Vero cells) WIBP COVID-19 vaccine	Sinopharm; China National Biotec Group Co; Wuhan Institute of Biological Products	2	Phase 4	NCT05065892			✓	✓	✓
				NCT05075044			✓	✓	✓
				NCT05075057				✓	✓
				NCT04790851			✓	✓	✓
				NCT04863638			✓	✓	✓
				NCT05065879					✓
				NCT05075083			✓	✓	✓
ChAdOx1-S (AZD1222) Covishield Vaxzevria	AstraZeneca + University of Oxford	1–2	Phase 4	NCT04760132			✓	✓	✓
				NCT04775069		✓		✓	✓
				EUCTR2021-002327-38-NL				✓	✓
				NCT04914832			✓	✓	✓
				ACTRN12621000661875		✓	✓	✓	✓
				RBR-4vmh6xg			✓	✓	✓
				EUCTR2021-004419-14-FI					✓
			phase 3	NCT04864561				✓	✓
				NCT04756271					✓
				NCT04536051	✓		✓	✓	✓
Recombinant novel coronavirus vaccine (adenovirus type 5 vector) Ad5-nCoV	CanSino Biological Inc./Beijing Institute of Biotechnology	1	Phase 4	NCT04892459			✓	✓	
				NCT05303584				✓	✓
Recombinant COVID-19 vaccine (adenovirus type 5 vector) for inhalation (Ad5-nCoV-IH)	CanSino Biological Inc./Beijing Institute of Biotechnology	1	Phase 4	NCT05303584	✓			✓	✓
			Phase 3	NCT05169008				✓	✓
				NCT05204589				✓	✓
Gam-COVID-Vac Adeno-based (rAd26-S + rAd5-S); Sputnik V COVID-19 vaccine	Gamaleya Research Institute; Health Ministry of the Russian Federation	2	Phase 3	NCT04530396	✓			✓	✓
				NCT04564716				✓	✓
				CTRI/2022/04/041792				✓	
				NCT04642339				✓	✓
				NCT04656613				✓	✓
				NCT04741061					✓
				PACTR202104601572565			✓	✓	✓
Ad26.COV2.S	Janssen Pharmaceutical (Johnson & Johnson)	1–2	Phase 4	EUCTR2021-002327-38-NL	✓		✓	✓	✓
				NCT05037266	✓		✓	✓	✓
				NCT05075538	✓	✓	✓	✓	
			Phase 3	NCT04614948			✓	✓	✓
				NCT05047640	✓		✓	✓	
				NCT05091307				✓	✓
				NCT05142319				✓	✓
				PACTR202102855526180				✓	✓
SARS-CoV-2 rS/matrix M1-adjuvant; full length recombinant SARS CoV-2 glycoprotein nanoparticle vaccine adjuvanted with matrix M; NVX-CoV2373	Novavax	2	Phase 3	NCT04611802			✓	✓	✓
				NCT05463068				✓	✓
				EUCTR2020-004123-16-GB			✓	✓	✓
				CTRI/2022/04/042017				✓	✓
				NCT05556720		✓		✓	✓
				NCT05249816				✓	✓
				NCT05372588				✓	✓
				NCT04583995	✓		✓	✓	✓
mRNA-1273; Spikevax	Moderna + National Institute of Allergy and Infectious Diseases (NIAID)	2	Phase 4	NCT04760132			✓	✓	✓
				NCT04885907				✓	✓
				EUCTR2021-002327-38-NL	✓	✓	✓	✓	✓
				EUCTR2021-003388-90-NL				✓	✓
				EUCTR2021-001202-30-NL				✓	✓
				NCT04952402			✓	✓	✓
				EUCTR2021-003618-37-NO	✓	✓	✓	✓	
				NCT05030974					✓
				NCT05047718					✓
				NCT05079633				✓	✓
				NCT05075538			✓	✓	✓
				EUCTR2021-004419-14-FI				✓	✓
			Phase 3	NCT05387317			✓	✓	✓
BNT162b2 (3 LNP-mRNAs), also known as Comirnaty	Pfizer/BioNTech + Fosun Pharma	2	Phase 4	NCT04760132				✓	✓
				NCT04780659					✓
				NCT04775069				✓	✓
				EUCTR2021-000893-27-BE		✓	✓		✓
				EUCTR2021-000930-32-BE				✓	✓
				RBR-4vmh6xg			✓	✓	✓
				NCT04852861			✓		✓
				NCT04878211				✓	✓
				EUCTR2021-003618-37-NO	✓		✓	✓	
				NCT04952766					✓
				NCT04969250					✓
				NCT05057169				✓	✓
				NCT05160766	✓			✓	✓
			Phase 3	NCT05405283				✓	✓
Recombinant SARS-CoV-2 vaccine (CHO Cell); Zifivax (ZF2001)	Anhui Zhifei Longcom Biologic Pharmacy Co., Ltd.	2–3	Phase 3	NCT05091411				✓	
				NCT04646590			✓	✓	✓
				NCT05128643					✓
CVnCoV vaccine	CureVac AG	2	Phase 3	NCT04674189			✓	✓	✓
				NCT04838847				✓	✓
				NCT04860258				✓	✓
SARS-CoV-2 vaccine (Vero cells)	Institute of Medical Biology + Chinese Academy of Medical Sciences	2	Phase 3	NCT04659239				✓	✓
				NCT05163652				✓	✓
				NCT05033847			✓	✓	✓
				NCT05164731				✓	✓
QazCovid-in®; COVID-19 inactivated vaccine	Research Institute for Biological Safety Problems, Rep of Kazakhstan	2	Phase 3	NCT04691908				✓	✓
INO-4800+electroporation	Inovio Pharmaceuticals + International Vaccine Institute + Advaccine (Suzhou) Biopharmaceutical Co., Ltd	2	Phase 3	ISRCTN15779782			✓	✓	✓
AG0301-COVID19	AnGes + Takara Bio + Osaka University	2	Phase 2/3	NCT04655625				✓	✓
nCov vaccine	Zydus Cadila	3	Phase 3	CTRI/2020/07/026352				✓	✓
BBV152 vaccine; COVAXIN	Bharat Biotech International Limited	2	Phase 3	NCT04641481			✓	✓	✓
VAT00008: SARS-CoV-2 S protein with adjuvant	Sanofi Pasteur + GSK	2	Phase 3	PACTR202011523101903	✓		✓		✓
				NCT05124171					✓
				NCT05405283				✓	✓
				NCT04904549	✓		✓		✓
Inactivated SARS-CoV-2 vaccine (Vero cells)	Shenzhen Kangtai Biological Products Co., Ltd.	2	Phase 3	NCT04852705			✓	✓	✓
GRAd-COV2; replication defective simian adenovirus (GRAd) encoding S	ReiThera + Leukocare + Univercells	1	Phase 2/3	NCT04791423		✓	✓	✓	✓
CpG 1018/alum-adjuvanted recombinant SARS-CoV-2 trimeric S-protein subunit vaccine (SCB-2019)	Clover Biopharmaceuticals Inc./Dynavax	2	Phase 3	NCT05012787	✓		✓	✓	✓
MVC-COV1901; spike-2P protein + adjuvant CpG 1018; MVC-COV1901(Beta)	Medigen Vaccine Biologics + Dynavax + National Institute of Allergy and Infectious Diseases	2	Phase 4	NCT05079633					✓
				NCT05097053			✓		✓
			Phase 3	NCT05198596			✓	✓	✓
FINLAY-FR-2 anti-SARS-CoV-2 vaccine (RBD chemically conjugated to tetanus toxoid plus adjuvant) Soberana 02	Instituto Finlay de Vacunas	2	Phase 3	RPCEC00000354			✓	✓	✓
EpiVacCorona (EpiVacCorona vaccine based on peptide antigens for the prevention of COVID-19)	Federal Budgetary Research Institution State Research Center of Virology and Biotechnology “Vector”	2	Phase 3	NCT04780035	✓	✓	✓	✓	✓
RBD (baculovirus production expressed in Sf9 cells); recombinant SARS-CoV-2 vaccine (Sf9 cells)	West China Hospital + Sichuan University WestVac Biopharma Co., Ltd.	2	Phase 3	NCT04887207			✓	✓	✓
				NCT04904471	✓		✓	✓	✓
UB-612 (Multitope peptide based S1-RBD-protein based vaccine)	Vaxxinity	2	Phase 3	NCT05293665			✓		✓
DelNS1-2019-nCoV-RBD-OPT1 (Intranasal flu-based-RBD)	University of Hong Kong, Xiamen University and Beijing Wantai Biological Pharmacy	2	Phase 3	ChiCTR2100051391			✓		✓
				PACTR202110872285345			✓		
SARS-CoV-2 mRNA vaccine (ARCoV)	Academy of Military Science (AMS), Walvax Biotechnology and Suzhou Abogen Biosciences	2	Phase 3	NCT04847102			✓		✓
Coronavirus-like particle COVID-19 (CoVLP); MT-2766	Medicago Inc.	2	Phase 2/3	NCT04636697			✓	✓	✓
			phase 3	NCT05040789					✓
COVI-VAC	Codagenix/Serum Institute of India	1–2	Phase 3	ISRCTN15779782	✓			✓	✓
CIGB-66 (RBD + aluminum hydroxide)	Center for Genetic Engineering and Biotechnology (CIGB)	3	Phase 3	RPCEC00000359	✓		✓	✓	✓
VLA2001	Valneva + National Institute for Health Research, UK	2	Phase 3	NCT04864561				✓	✓
				NCT04956224				✓	✓
BECOV2	Biological E. Limited	2	Phase 3	CTRI/2021/08/036074				✓	✓
Recombinant SARS-CoV-2 spike protein, aluminum adjuvanted (Nanocovax)	Nanogen Pharmaceutical Biotechnology	2	Phase 3	NCT04922788			✓	✓	✓
Recombinant protein vaccine S-268019 (using baculovirus expression vector system)	Shionogi	2	Phase 3	NCT05212948			✓		
				JPRN-jRCT2031210613					✓
TURKOVAC; inactivated virus	Erciyes University and the Health Institutes of Turkey (TUSEB)	2	Phase 3	NCT04942405					✓
				NCT05077176				✓	✓
GBP510, a recombinant surface protein vaccine with adjuvant AS03 (aluminum hydroxide)	SK Bioscience Co., Ltd. and CEPI	2	Phase 3	NCT05007951				✓	✓
Razi Cov Pars, recombinant spike protein	Razi Vaccine and Serum Research Institute	3	Phase 3	IRCT20210206050259N3			✓	✓	✓
COVID-19 inactivated vaccine, (CovIran-Barkat)	Shifa Pharmed Industrial Co	2	Phase 2/3	IRCT20201202049567N3			✓	✓	✓
ReCOV: recombinant two-component spike and RBD protein COVID-19 vaccine (CHO cells)	Jiangsu Rec-Biotechnology	2	Phase 3	NCT05398848			✓	✓	✓
ABNCoV2 capsid virus-like particle (cVLP) ± adjuvant MF59	Radboud University	2	Phase 3	NCT05329220				✓	✓
Recombinant SARS-CoV-2 fusion protein vaccine (V-01)	Livzon Pharmaceutical	2	Phase 3	NCT05096832	✓		✓	✓	✓
				NCT05096845	✓		✓	✓	✓
Inactivated COVID-19 vaccine	KM Biologics Co., Ltd.	2	Phase 3	JRCT2031210679				✓	
RBD protein recombinant SARS-CoV-2 vaccine (Noora vaccine)	Bagheiat-allah University of Medical Sciences/AmitisGen	3	Phase 3	IRCT20210620051639N3					✓
Recombinant protein RBD fusion dimer adjuvanted vaccine; COVID-19 vaccine Hipra; PHH-1V	Laboratorios Hipra, S.A.	2	Phase 3	NCT05246137	✓	✓	✓	✓	✓
				NCT05303402			✓	✓	
ARCT-154 mRNA Vaccine	Arcturus Therapeutics, Inc.	2	Phase 3	ISRCTN15779782	✓			✓	✓
SCTV01C; bivalent recombinant trimeric S protein vaccine against SARS-CoV-2 variants	Sinocelltech Ltd.	1	Phase 3	NCT05308576			✓		✓
				NCT05323461				✓	✓
Inactivated whole virion concentrated purified vaccine (CoviVac)	Chumakov Federal Scientific Center for Research and Development of Immune-and-Biological Products	2	Phase 3	NCT05407142			✓		✓

NCT, clinicaltrials.gov (https://clinicaltrials.gov/ct2/home); IRCT, Iranian Registry of Clinical Trials (https://en.irct.ir/); ChiCTR, Chinese Clinical Trial Registry (https://www.chictr.org.cn/searchprojen.aspx); EUCTR, European Union Clinical Trials Register (https://www.clinicaltrialsregister.eu/ctr-search/search); ANZCTR, Australian New Zealand Clinical Trials Registry (https://anzctr.org.au/Default.aspx); PACTR, Pan African Clinical Trials Registry (https://pactr.samrc.ac.za/Search.aspx); ISRCTN, International Standard Randomized Controlled (https://www.isrctn.com); CTRI, Clinical Trials Registry-India (http://ctri.nic.in/Clinicaltrials/login.php); RPCEC, Cuban Public Registry of Clinical Trials (https://rpcec.sld.cu/trials); JPRN, Japan Primary Registries Network (https://rctportal.niph.go.jp/en/link); JRCT, Japan Registry of Clinical Trials (https://jrct.niph.go.jp/); RBR, Brazilian Registry of Clinical Trials (https://ensaiosclinicos.gov.br/).

## SARS-CoV-2 reinfection

One of the largest enigmas surrounding COVID-19 is the probability of reinfection and/or reactivation of latent viral hideouts in the body, as well as the potential negative effects of some drugs on the formation of acquired immunity to the virus. These questions can be considered central for biomedical scientists fighting the outbreak of SARS-CoV-2.^288^ Until nearly the third month of the COVID-19 pandemic, scientists expected that the disease would be non-relapsing and would be eliminated through immunization, such that all recovered patients would gain sufficient acquired immunity to SARS-CoV-2. However, as the number of patients with COVID-19 increased, some recovered patients reported relapse of COVID-19 signs and symptoms after they were completely cured and discharged from hospitals after displaying repeatedly positive SARS-CoV-2 RT-PCR tests. Hence, the dilemma of COVID-19 reinfection versus acquired immunity to SARS-CoV-2 has been raised and requires further research.

The first report showing a positive SARS-CoV-2 RT-PCR assay for a convalescent case (after two consecutive negative results) was from China.^289^ Another case report described three patients with COVID-19 who were readmitted in March 2020 to a hospital in Wuhan, China, after having recovered with positive SARS-CoV-2 IgG antibody, negative SARS-CoV-2 RT-PCR and negative SARS-CoV-2 IgM antibody tests.^290^ Those patients were readmitted because of positive SARS-CoV-2 RT-PCR results after convalescence, although they did not display any of the characteristic COVID-19 signs or symptoms again, and the results of their IgG and IgM tests remained stable.^290^ Thereafter, in April 2020, a total of 116 COVID-19 survivors from South Korea tested positive with SARS-CoV-2 RT-PCR tests after recovery.^291^ These positive tests were confirmed according to the Korean criteria, wherein patients must have two consecutive SARS-CoV-2 negative samples within 24 h to be considered to have recovered from COVID-19.^292^ In these two reports, the possibility of reinfection was excluded because of the absence of evidence that the detected SARS-CoV-2 RNA was from replication-competent viruses that could be contagious.

Moreover, a case report has described 11 patients with COVID-19 from France in April and May 2020, who experienced second separate symptomatic SARS-CoV-2 infections after recovery, as confirmed by RT-PCR.^288^ In May 2020, another case of possible COVID-19 reinfection emerged in the USA.^9^ In that case, 1 week after confirmed recovery and discharge, the patient was readmitted with both radiologically and virologically (RT-PCR) confirmed reinfection.^9^ Although two similar cases have been reported in South Korea,^293^ none of these reports have confirmed whether the convalescence was due to viral reactivation from a hideout in the body or to viral reinfection.

In a retrospective study of 55 patients with COVID-19 who had been admitted to a hospital in Wuhan, five were readmitted after recovery and discharge.^294^ All five patients returned with confirmed SARS-CoV-2 infection, as diagnosed on the basis of clinical, laboratory and CT examinations after 4–17 days; the authors hypothesized that SARS-CoV-2 reactivation was the reason.^294^ The first SARS-CoV-2 reactivation case in Italy was reported 2 weeks after primary infection; the patient was cured, as confirmed by radiology and two consecutive RT-PCR tests. Interestingly, the results indicated the presence of SARS-CoV-2 IgG antibodies in the patient's serum, but not IgM, thus revealing that his immune system was beyond the acute phase of COVID-19.^295^

To investigate the scope of acquired immunity to SARS-CoV-2, another early study conducted by researchers from China infected Rhesus macaques with SARS-CoV-2, then reinfected them after confirmed recovery.^296^ Viral replication was detected (nasopharyngeal and anal swabs), and COVID-19 symptoms were recorded in the primary infection, and neutralizing activity against SARS-CoV-2 was confirmed after recovery. However, the viral replication and signs of COVID-19 were not detected after reinfection, thus suggesting that acquired immunity to SARS-CoV-2 may protect against reinfection.^296^ However, how long the acquired immunity to SARS-CoV-2 lasts is uncertain. A study on individuals infected with COVID-19, for example, has indicated a consistent decrease in neutralizing SARS-CoV-2 antibody levels over a 2–3 month period following recovery.^297^

These early studies have clarified that reinfection with SARS-CoV-2 is a realistic scenario and not a mere possibility. According to a recent study in Tunisia, reinfection has been detected in four women and HCWs 32–46 years of age.^8^ Whole-genome sequencing of the viral RNA was performed on the clinical specimens that retested positive for SARS-CoV-2 by RT-PCR after recovery from the first infection and also on samples collected during the initial infection. A reinfection episode was detected in all four patients. Through comparative genome analysis, unique viral lineages were detected among the four patients.^8^ The duration between infection episodes ranged between 45 and 141 days, and symptoms were milder in two patients during the second infection and more severe in the other two patients. Despite the presence of antibodies in three of the patients, all four patients had reinfection.^8^

However, Lumley and colleagues have reported no symptomatic reinfections in HCWs who had anti-spike antibodies, thus indicating that previous SARS-CoV-2 infection may result in anti-spike antibodies that protect against reinfection in most people, at least for the first 6 months.^298^ Recently, in contrast to that after two rounds of vaccination, the IgG response in COVID-19 infected people has been demonstrated to be characterized by insufficient avidity maturation. Insufficient avidity maturation may promote SARS-CoV-2 reinfection and consequently impede the development of herd immunity in susceptible individuals.^299^^,^^300^ Remarkably, the conjunction of natural infection and one step of vaccination resulted in the same degree of high IgG avidity as two rounds of vaccination, hence increasing the protective immune response.^300^ According to these findings, individuals who have been exposed to different COVID-19 strains may not be able to develop sufficiently protective immune responses through natural infection, thus highlighting the importance of vaccination and regular monitoring of their immune status, both qualitatively and quantitatively.

A prospective cohort study has recently been conducted among staff at the International Centre for Diarrheal Disease Research in Bangladesh, who had been registered for COVID-19 testing by real-time RT-PCR.^7^ In this study, 750 positive patients were identified to have reinfection, 27.6% of whom required hospitalization and 2.5% of whom died. Among the positive cohort, 731 patients, excluding those who died, who tested positive for COVID-19 (episode 1) were followed up for 0.91 person-years, and 38 (5.7 reinfections/100 person-year) were identified as having reinfection (episode 2). The probability of infection was 80.2% lower in the positive cohort than the negative cohort. Genome sequencing revealed that reinfections were caused by genetically different COVID-19 strains. Naturally infected populations had a lower risk of reinfection with COVID-19 than vaccinated and infection-naive groups. Additionally, the lower mean Ct-value detected among symptomatic patients than asymptomatic patients suggested that the development of COVID-19-like symptoms and seroconversion were due to either a low Ct value or a high viral load.^7^ Although reinfected patients did not develop severe disease, a substantial number of naturally infected or vaccinated people were more likely to be reinfected by emerging strains.

Moreover, another prospective cohort study has reported that among 2625 patients who had at least one SARS-CoV-2 infection across the 10-month study period, 5.94% experienced reinfection, and 20.57% experienced recurrence following the first infection.^301^ The average number of days to reinfection was 126.50 days, whereas the average number of days to recurrence was 31.50 days. The incidence rate of reinfection and recurrence was 0.35 cases/1000 person-days and 1.47 cases/1000 person-days, respectively, and most recurrences was detected within 30 and 60 days after the first infection. Moreover, people working in clinical units were at greater risk of reinfection than those working in non-clinical facilities.^301^ This study contributes to the growing consensus that COVID-19 reinfection, defined as second infection >90 days after initial infection, is uncommon, even among a HCWs who are regularly exposed to the virus. In addition, this study has emphasized the urgent need for widespread vaccination and booster doses to alleviate public health costs.

Although the current vaccines have been demonstrated to be less effective against one or more variants than the original strain, studies have shown that full vaccination is reasonably effective in preventing COVID-19 associated hospitalization and death among vaccinated people compared with non-vaccinated people.^7^^,^^260^^,^^302^, ^303^, ^304^, ^305^, ^306^ Studies have demonstrated that Pfizer-BioNTech or Moderna vaccination effectively provides cross-variant neutralization against both the Beta (B.1.351) and Alpha variants following a natural infection by boosting the responses of neutralizing antibody elicited by COVID-19 variants.^307^^,^^308^ According to these findings, complete vaccination against COVID-19 confers additional protection for people with a previous SARS-CoV-2 infection. In contrast, the molecular evidence for reinfection has indicated that genetically different strains or novel variants can evade the immune response regardless of whether they were acquired from vaccination or natural infection.^309^ In a study by Tan et al., all patients, even those with minimal symptoms, exhibited a cellular response to COVID-19 antigens, thus providing cause for optimism in terms of preventing reinfections.^310^ In contrast, some authors have postulated mechanisms explaining severe second reinfections, including a larger viral load and acquiring a more pathogenic variant.^311^

In addition, several studies have found lower vaccination effectiveness against the Delta (B.1.617.2) variant than against the Alpha (B.1.1.7) or Wuhan (B.1) variants.^41^, ^312^, ^313^ One of these studies, conducted in Massachusetts, has reported that more than 74% of 469 patients who were predominantly affected by the Delta variant had been fully vaccinated.^314^ Therefore, full understanding of immunity against SARS-CoV-2 infection is urgently needed, to determine the likelihood of second infection and the longevity of vaccine protection. Consequently, assessing the vaccine's effectiveness against newly emerging strains will be critical.

At this point in the discussion, an etymological clarification of the terms “reinfection” and “recrudescence/reactivation” should be made. These two terms have similar outcomes (i.e., viral infection and outbreak) but differ in the origin/cause. In the case of reinfection, recovered patients are susceptible to infection again by an identical or a new strain of the virus. In contrast, in reactivation, although they are immunologically protected, recovered patients are susceptible to the reappearance of disease symptoms due to the persistence of the virus.^315^ To further understand the underlying mechanisms in cases of COVID-19 reinfection or reactivation, researchers have highlighted the importance of the assessment of adaptive immunity to SARS-CoV-2, and of the genomic comparison of SARS-CoV-2 strains in both episodes.^288^

In general, the occurrence of SARS-CoV-2 reactivation has been suggested to depend on three key risk factors: health status of the host, virological factors (e.g., the viral load and the mutation rate) and whether the host is taking an immunosuppressive medication.^294^ Notably, the false-negative RT-PCR results can be obtained due to a low viral load and technical or human errors.^289^ False-positive results after recovery can also occur because of the recurrence of SARS-CoV-2 RNA fragments as a result of the detoxification process, or because of human or technical errors during sampling, transport, analysis and recording.^316^

In contrast, because the duration of RT-PCR positivity may vary among patients,^317^ viral replication may persist for longer periods than expected, thus resulting in late reactivation. Questions regarding whether SARS-CoV-2 can remain active for longer periods than expected, or can subside and reactivate again, lack clear answers, thus increasing the possibility of silent carrier status development.^316^ Therefore, patients with COVID-19 must crucially be followed during the convalescence period, with a commitment of 14 days quarantine post-discharge. These cases highlight the importance of continuous surveillance of the results of SARS-CoV-2 molecular tests (i.e., quantitative RT-PCR) for the possibility of reinfection and infectivity assessment in patients at convalescence. In addition, the probability of reinfection by the same strain or another genetically mutated strain should be considered.

In addition to the paucity of reports addressing the reinfection dilemma, these reports did not perform crucial comparisons between the genome sequences of SARS-CoV-2 in primary infection and reinfection, and thus could not confirm whether the detected cases of convalescence were due to reinfection or reactivation. Because RT-PCR detects the virus on the basis of a specific small sequence of the viral genome rather than its whole sequence, the test cannot differentiate between reactivation and reinfection, nor can they indicate which strain has caused the reinfection. With this in mind, researchers from the University of Hong Kong have recently followed a case of a confirmed second episode of SARS-CoV-2 infection after more than 4 months of recovery^318^ and performed a genomic comparison of SARS-CoV-2 genome sequences in both episodes. Interestingly, the genome sequence of the SARS-CoV-2 in the second episode of infection significantly differed from that in the first episode.^318^ Therefore, this report has suggested that COVID-19 convalescence is due to reinfection, possibly because SARS-CoV-2 is a positive-sense RNA virus with a comparatively high mutation rate. In the environment, the variability of SARS-CoV-2 is likely to resemble that of the common cold, another RNA virus in constant circulation. Although SARS-CoV-2 may share the high transmissibility and high asymptomatic rates of its relatively benign genetic relative, it is bound to have a far higher fatality rate than the latter.

**Figure 6:**
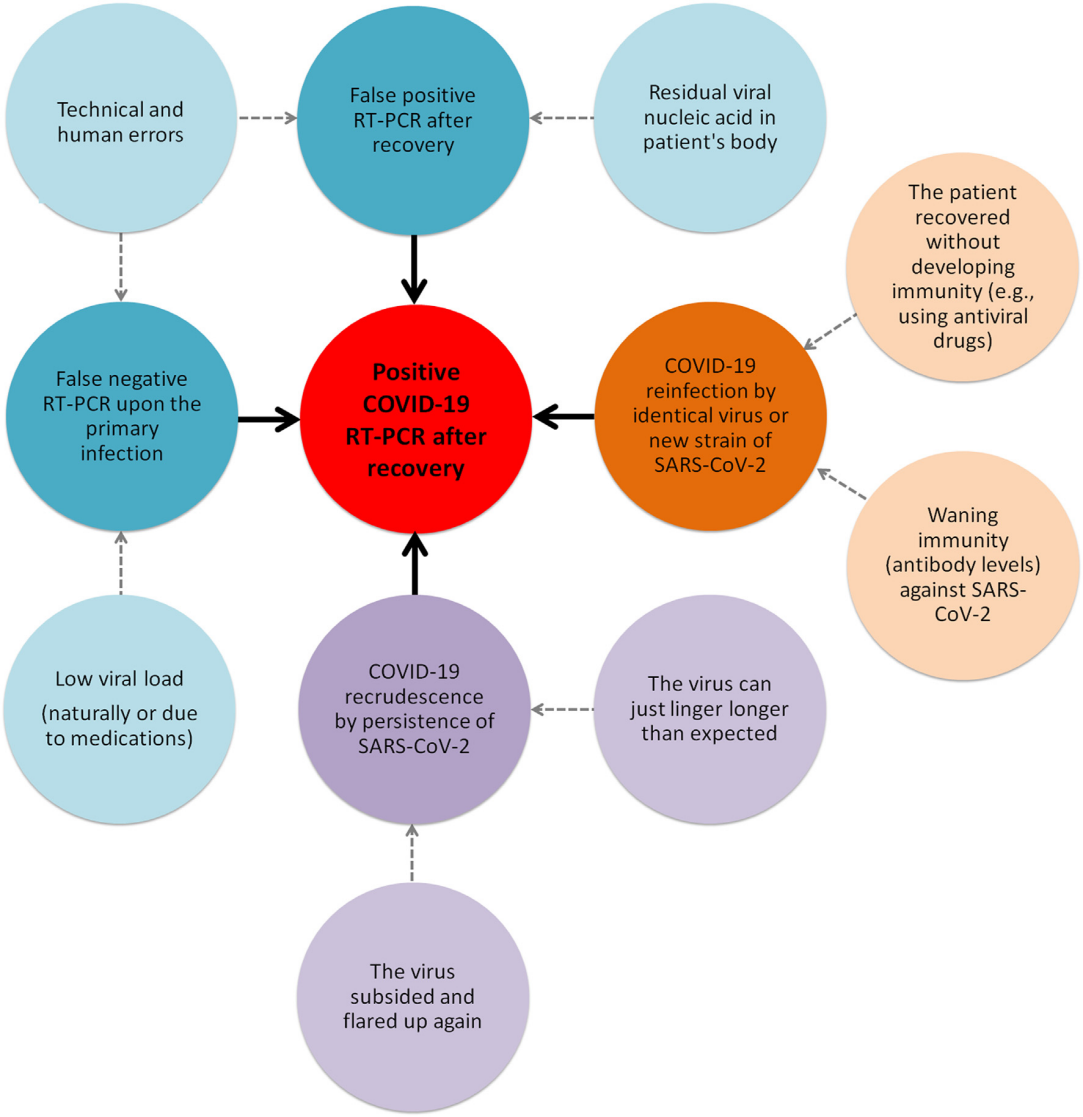
Illustration of potential explanations for positive SARS-CoV-2 RT-PCR tests in recovered patients with COVID-19.

According to the data available to date, the potential explanations for the positive SARS-CoV-2 RT-PCR results in COVID-19 survivors can be summarized as shown in Figure 6.

## Future directions

As SARS-CoV-2 variants have continued to evolve and spread, the risk of COVID-19 reinfection has grown. Hence, caution should be exercised even after confirmed recovery, particularly in older people who are more susceptible to severe COVID-19, and for those with preexisting chronic diseases and/or pneumonia. To ensure a complete cure and prevent recurrence, post-recovery monitoring and stratified management should be implemented whenever possible. Reports on COVID-19 reinfection are limited, owing to the negligible number of these reports and their small sample sizes. Given the global emergency caused by this pandemic, comprehensive studies involving further immunological, virological and epidemiological data are needed to provide further understanding of the underlying pathophysiology of SARS-CoV-2 reinfection.

Owing to the dramatic increase in the number of both active and recovered cases globally, the issue of COVID-19 reinfection has been brought to the public's attention as a necessary factor to consider to properly predict the spread of SARS-CoV-2 and its expected pandemic trajectory. On the basis of the literature discussed above, both herd immunity and vaccines may not be able to eliminate COVID-19 and establish permanent immunity against SARS-CoV-2. Given the cost and time required to develop herd immunity and vaccines, scientists must seek further valuable and sustainable solutions requiring less time and cost rather than being satisfied with the current approaches.

## Conclusions

The current approval of several vaccines for COVID-19 has given the global population hope that the pandemic will be overcome. The urgency of the pandemic, the technological advances and existing vaccine candidates have contributed to the rapid development of vaccines for COVID-19. Nevertheless, much about COVID-19 and its causative agent, SARS-CoV-2, remains undiscovered, including the human response to vaccines and the emergence of reinfection or reactivation of infection, thus further complicating understanding of COVID-19. Hence, healthcare practices and the scientific community should be made aware of these issues for devising guidelines and future R&D plans targeted to the eradication of the COVID-19 pandemic.

## Source of funding

This work was supported by a grant from the 10.13039/501100003093Ministry of Higher Education, Malaysia (grant number: FRGS/1/2020/SKK06/USM/03/2).

## Conflict of interest

The authors have no conflict of interest to declare.

## Ethical approval

Not applicable.

## Authors contributions

MAIA-H and RM conceived and designed the study, and conducted the preliminary review of articles. MAIA-H, MAHA, MHM-Z and ENSEAR determined the research scope, and collected and organized the extracted data. MAIA-H and MAHA analyzed and interpreted the data. MAIA-H, MAHA, MHM-Z, ENSEAR, MMH, WA, SA, CYY, IZA, VU and RM wrote the initial and final drafts of the article, and provided logistic support. All authors have critically reviewed and approved the final draft and are responsible for the content and similarity index of the manuscript.

## Acknowledgment

Both MAIA-H and ENSEAR would like to acknowledge the 10.13039/501100004595Universiti Sains Malaysia (USM) Fellowship Scheme for providing financial support. MHM-Z would like to acknowledge the 10.13039/501100003971Islamic Development Bank (IsDB) for providing financial support under the PhD Scholarship Program.
